# Supplementary material for: Real-time single-molecule imaging of CaMKII-calmodulin interactions
Source: Biophys J. 2024 Feb 28;123(7):824–38. doi: 10.1016/j.bpj.2024.02.021 (PMC11630639; doi:10.1016/j.bpj.2024.02.021)
Supplement: Document S1. Figures S1 and S2 and Table S1 [file mmc1.pdf]

Biophysical Journal, Volume *123*

Supplemental information

Real-time single-molecule imaging of CaMKII-calmodulin interactions

Shahid Khan, Justin E. Molloy, Henry Puhl, Howard Schulman, and Steven S. Vogel

#	Construct	Primer (Set)	Primer (Name)	Primer (5'-3' Sequence)
1	6His-SUMO-V-rCaMKIIβ*	Vector set:	SATVLBetaIFfor:	CGAGCTCAAGCTTCGAATTCTGCAGTCGACGGTTTAAATGATATCTTTGAAGC
			SATIFrev:	TCCACCAATCTGTTCTCTGTG
		Insert set:	VenforIF:	CCGCGCGGCAGCCATGTGAGCAAGGGCGAGGAGCTG
			VC1LinkIFrev:	CGAAGCTTGAGCTCGAGATC
2	6His-SUMO-V15-mCaMKIIα*	Vector set:	SMT3forIF:	GGATCCACCTCGTCCAATGCAG
			SATIFrev:	TCCACCAATCTGTTCTCTGTG
		Insert set:	ATVC1IFfor:	GAACAGATTGGTGGAGTGAGCAAGGGCGAGGAGC
			VAlpharevIF:	GGACGAGGTGGATCCTCAATGCGGCAGGACGGAG
3	6His-SUMO-V15-rCaMKIIβ**	Deletion set:	VBlinkerModFor:	GCCACCACAGTGACCTGCACC
			VBlinkerModRev:	GTCGACTGCAGAATTCGAAGC
4	6His-V15-rCaMKIIβ*	Deletion set:	VenforIF:	CCGCGCGGCAGCCATGTGAGCAAGGGCGAGGAGCTG
			SMT3revIF:	ATGGCTGCCGCGCGGCACCAG
5	6His-V15-rCaMKIIβ(monomeric/Δ315)**	Deletion set:	rCK2QC315F:	**TGA**TGCCCCGGTGGCCCCGTTACAG
			rCK2QC315R:	**TTA**CACACTGAAATTACGGGTGGC
6	6His-V15-rCaMKIIβ(dimeric/F458A)**	Mutagenesis	ratF458Afor:	**GCC**CACCGTTTCTATTTTGAAAACCTGC
			ratF458Arev:	GTCCATACCCTCCACCAGATTACC
7	6His-V15-mCaMKIIα(dimeric/F394A)**	Mutagenesis	mouseF394Afor:	**CGC**CATCGATTCTATTTTGAAAACTTG
			mouse394Arev:	GTCCAGGCCCTCCACCAGGTTC
8	6His-V15-rCaMKIIβ(silent/T287A)**	Mutagenesis	ratT287Afor:	**GCG**GTGGAATGCCTGAAGAAGTTTAATGC
			ratT287Arev:	CTCCTGGCGATGCATCATGCTTG
9	6His-V15-rCaMKIIβ(T287A/T306A/T307A)**&	Mutagenesis	ratT306/307Afor:	**GCTGCG**ATGCTGGCCACCCGTAATTTCAG
			ratT306/307Arev:	CAGAATGGCGCCCTTTAACTTGC
10	6His-V15-rCaMKIIβ(phosphomimic/T287D)**	Mutagenesis	ratT287Dfor:	**GAT**GTGGAATGCCTGAAGAAGTTTAATGC
			ratT287Drev:	CTCCTGGCGATGCATCATGCTTG
11	6His-V15-rCaMKIIβ(ATP-binding/K43R)**	Mutagenesis	βK43Rfor	**CGC**ATTATTAACACCAAGAAACTGAGC
			βK43Rrev	GGCGGCATATTCGTGGCCTGTAC

Table S1: Plasmid constructions. *[*]The In-Fusion Snap Assembly cloning system (TaKaRa Bio-USA, San Jose, CA) was used for insertions and some deletions. Larger insertions required amplification of overlapping vectors and insert PCR products as in constructs 1 and 2. Deletions required the generation of a single PCR product with overlapping ends as in construct 3-5, 10 and 11. [**]Q5® Site-Directed Mutagenesis Kit (New England Biolabs. Ipswich, MA) was used for all site-directed mutagenesis reactions and the deletion reaction in construct 3. [&]Mutagenesis was performed sequentially using construct 8 as the template for the triple mutant T287.306-307A. The rat and mouse CaMKII-ADs have 100% sequence identity.*

A

ASSEMBLY	[CaM]nM	[ATP]	CC_{MEAN}	CC_{SE}
β-Monomer	30	(-)	0.07	0.01
	300	(-)	0.12	0.005
	300	(+)	0.14	0.02
β-Dimer	30	(+)	0.2	0.1
	300	(+)	0.26	0.1
α-Dimer	30	(-)	0.04	0.01
	3	(+)	0.13	0.05
	30	(+)	0.34	0.1
	300	(+)	0.45	0.03
β-Holoenzyme	300	(-)	0.1	0.01
	30	(+)	0.59	0.04
	100	(+)	0.79	0.01
α-Holoenzyme	3	(+)	0.3	0.05

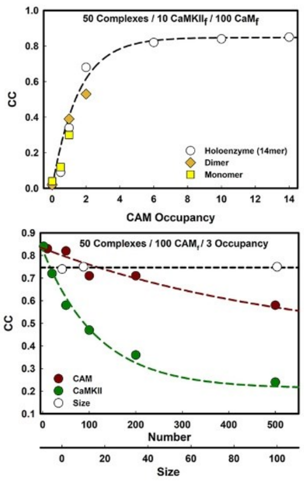

D

Construct	ATP	[r-CAM]nM	f_{ON}	f_{OFF}	$k_{\tau1}$	$k_{\tau2}$	$k_{\tau1\text{-}corr}$	A*i* nM
βmon	(-)	300	0.05	0.79	0.69			4140
βmon	(+)	300	0.042	0.79	0.69			4928.6
αdim	(+)	300	0.074	0.19	0.155			628.4
αdim	(+)	30	0.032	0.46	0.59	0.03		553.1
βdim	(+)	30	0.071	0.41	0.53			223.9
βholo	(+)	30	0.0455	0.074	0.24		0.315(0.06)	158.2

Figure S1: A. Table. Colocalization at different r-CAM concentrations (+/- ATP). *The $CC_{G\text{-}R}$ values measured colocalization. Each $CC_{G\text{-}R}$ value was based on at least 2 independent experiments, with >10 records per experiment. The typical record duration was 40 seconds (400 frames) with 30-50 spots per frame.* ***B. Figure. Simulation of $CC_{G\text{-}R}$ as a function of r-CAM occupancy.*** *The CaMKII holoenzyme tetradecamer (circle), dimer (diamond) and monomer (square). The occupancy saturates at 2 and 1 for the dimer and monomer respectively.* ***C. Figure. Simulation of $CC_{G\text{-}R}$ as a function of population size.*** *Free V-CaMKII (green circles), r-CAM (red circles) molecules and total population size (open circles). Three values for population size (1,10,100 x (50(CaMKII(14)/CAM(3) complexes), 10 free V-CaMKII, 100 free r-CAM).* ***D. Table. Apparent rate constants (s^{-1}).*** *The r-CAM concentration varied over the 1000 ->30 nM range. The monomer (βmon), dimer((αdim, βdim), and holoenzyme (βholo) assemblies. The WT β holoenzyme $k_{\tau1-corr}$ value is the photobleaching corrected $k_{\tau1}$ (Figure 4B). The photobleaching correction was not needed for the monomer or dimer assemblies. The step finder algorithm will detect spurious spots in the monomer records as the monomer intensity is not much different from background particulates and will miss ON events shorter than the algorithm's detection limit (0.5s). Both factors will underestimate f_{ON} and elevate A_i values.*

A

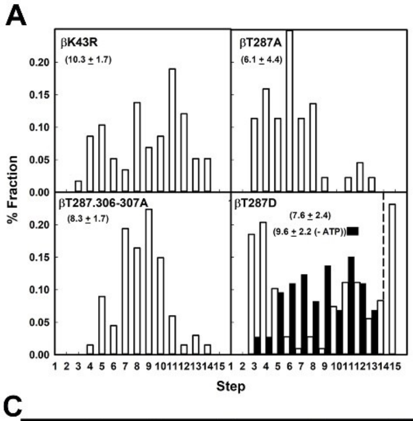

B

ASSEMBLY	[CaM]nM	[ATP]	CC_{MEAN}	CC_{SE}
β-T287A	6	(+)	0.1	0.05
	30	(+)	0.5	0.04
β-T287.306-307A	30	(+)	0.59	0.03
	30	(-)	0.44	0.03
β-K43R	3	(+)	0.53	0.05
	30	(+)	0.56	0.04
	30	(-)	0.15	0.05
β-T287D	0.03	(+)	0.62	0.07
	0.003	(+)	0.56	0.04
	0.3	(-)	0.78	0.09
	0.0003	(-)	0.75	0.04

C

PARAMETER	K43R	T287A	T287.306-307A	T287D(+)	T287D(-)	WT	WT_{CORR}
$M_{N\text{-}CAM}$	2.55±0.34	1.1±0.3	1.9±0.34	3.6±2.6	6.8±2.0	2.6±0.3	2.6±0.3
$M_{POISSON}$	2.14±0.17	1.7±0.16	1.45±0.03	3.0±0.25	6.2±0.35	0.9±0.22	2.4±1.6(98%)/8.2(2%)
R	0.95	0.95	0.99	0.9	0.82	0.85	0.93
$PEARSON_{CC}$	0.95	0.95	0.99	0.91	0.845	0.89	0.93

D

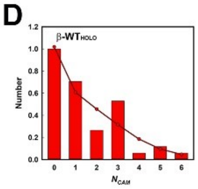

E

Construct	ATP	[r-CAM]nM	f_{ON}	f_{OFF}	$k_{\tau1}$	$k_{\tau2}$	$k_{\tau1\text{-}corr}$	*Ai* nM
βholo–K43R	(-)	30	0.035	0.94	3.18	0.45		2725.7
βholo-K43R	(+)	30	0.029	0.074	0.09		0.115(0.09)	93.1
βholo-T287A	(+)	30	0.033	0.5	0.53		0.655(0.12)	481.8
βholo-T287.306-307A	(+)	30	0.0165	0.055	0.092		0.11(0.07)	167.3
	(-)	30	0.057	0.11	0.26			136.8
βholo-T287D	(-)	0.3	0.105	0.112	0.11			
βholo-T287D	(-)	0.0003	0.056	0.148	0.15			
βholo-T287D	(+)	0.03	0.056	0.21	0.21			
βholo-T287D	(+)	0.003	0.04	0.1	0.095			

F

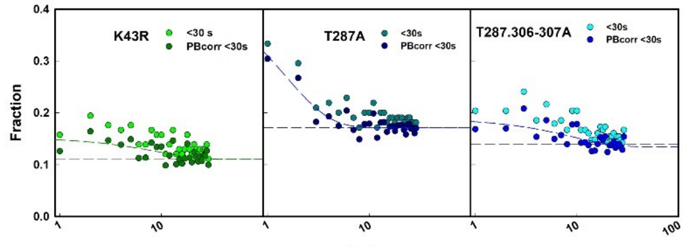

***Figure S2: A. V-CaMKII subunit stoichiometry distributions**. The $\overline{S_{CaMKII}}$ values are compared between populations. **B. Table of colocalization coefficients ($CC_{G\text{-}R}$).** The reported $CC_{G\text{-}R}$ values were based on independent experiments with the number and duration of records analyzed similar to that for the different assemblies (Figure S1A). **C. Table.** $M_{N\text{-}CAM} = \overline{N_{CAM}}$, $M_{POISSON}$ = Mean of Poisson distribution $N_{norm}^{Poisson} = \sum_{N_{CAM}=1}^{14}(N_{CAM}\,^{\wedge}M_{POISSON})(e^{-M_{POISSON}})/N_{CAM}!)$. R = Regression coefficient, P = Pearson correlation between the fit and observed values. WT_{corr} fit equation ($N_{norm}^{WTc0rr} = a.N_{norm}^{Poisson} + (1-a)\sum_{N_{CAM}=1}^{1}\left(e^{-(N_{CAM}-M_{POISSON}^{2})^{2}/M_{POISSON}^{2})}\right)$). **D. Plot.** Fit to the WT distribution(red bars) with the WT_{corr} fit equation. The two Poisson fit has three floating parameters: the mean of the first Poisson distribution ($M_{POISSON}$), the mean of the second Poisson (Gaussian) distribution ($M_{POISSON}^{2}$), and "a" the amplitude fraction of the first Poisson distribution. The goodness of fit for the T287D holoenzyme N_{CAM} distribution also improved when it was fit by a two-Poisson, but the physical significance of such a fit is not presently known. **E. Table. Apparent rates (s^{-1}).** The r-CAM concentration varied over the 30nM ->0.3 pM range. The β holoenzyme (K43R, T287A, T287.305-306A) $k_{\tau1-corr}$ values are the photobleaching corrected $k_{\tau1}$ rates. **F. Photobleaching corrected <30s |τ| distributions.** Single exponential fits. The N_τ values are expressed relative to the value of the $N_{\tau>30}$ fraction (0 -> dashed lines).*

APPENDIX-1: Abbreviations and Operations

1. Abbreviations

ABBREVIATIONS		PARAMETERS	
r-CAM	rhodamine-tagged Ca^{2+}.CAM	N_{CAM}	r-CAM cccupancy / CaMKII
V-CaMKII	Venus-tagged CaMKII subunit	S_{CaMKII}	CaMKII subunit stoichiometry
KD	CaMKII kinase domain	f_{ON}	Frequency - ON events
AD	CaMKII association domain	f_{OFF}	Frequency - OFF events
R	CaMKII regulatory segment	$\|\tau\|$	Modulus (residence time intervals)
*CC**	Cross-correlation coefficient	$\tau+$	Residence time intervals post ON event
P	Pearson coefficient	$\tau-$	Residence time intervals post OFF event
A_i	Affinity index	$k_{\tau*}$	Exponent from fit to $\|\tau\|$ distribution (*=order)

Table A1.1: Key. *All abbreviations are defined when first used. There are three CC*'s (*$CC_{G\text{-}R}$, $CC_{G\text{-}FRET}$, $CC_{R\text{-}FRET}$*). There are two k_{τ^*}'s ($k_{\tau 1}, k_{\tau 2}$) for biexponential fits to the $|\tau|$ distribution.*

2. Spot cross-correlations (ImageJ operations)

(a) Morphological kernels for spot detection.

The 7x7 Gaussian filter

```
var mGauss = "text1=[";
 mGauss = mGauss +  "1 1 1 1 1 1 1\n";
 mGauss = mGauss +  "1 2 2 2 2 2 1\n";
 mGauss = mGauss +  "1 2 4 5 4 2 1\n";
 mGauss = mGauss +  "1 2 5 6 5 2 1\n";
 mGauss = mGauss +  "1 2 4 5 4 2 1\n";
 mGauss = mGauss +  "1 2 2 2 2 2 1\n";
 mGauss = mGauss +  "1 1 1 1 1 1 1 ]";
```

The 9x9 Laplacian of Gaussian (LoG) filter

```
var LoG = "text1=[";
  LoG += " 0  0 -1 -1 -1 -1 -1  0  0\n";
  LoG += " 0 -1 -1 -1 -1 -1 -1 -1  0\n";
  LoG += "-1 -1 -1  0  1  0 -1 -1 -1\n";
  LoG += "-1 -1  0  4  7  4  0 -1 -1\n";
  LoG += "-1 -1  1  7 10  7  1 -1 -1\n";
  LoG += "-1 -1  0  4  7  4  0 -1 -1\n";
  LoG += "-1 -1 -1  0  1  0 -1 -1 -1\n";
  LoG += " 0 -1 -1 -1 -1 -1 -1 -1  0\n";
  LoG += " 0  0 -1 -1 -1 -1 -1  0  0 ]";
```

The top 1% of detected spots were tagged with unique numbers that applied over all three channels for each spot.

b) 2D-image cross-correlation operation

```
/* This macro assumes you have two, equal sized, 32-bit images
The perimeter pixel region is set to zero
The images are cross-correlated and normalised by co-variance

The Pearson Correlation Coefficient "r" (ranges from -1 to 1)
E.g. for cross-correlation of a red and a green image:

          sum { (green[x,y] - gmean) * (red[x,y] - rmean) }
     r = ----------------------------------------------------
               std_dev {green} * std_dev {red}

Perform calculations after relative shifting the red image past the green
image

Rectangular matrix (dx,dy) = up to 16 pixels still quite fast!
          1 pixel shift in "x" up to +/-dx then
          shift down 1 pixel in "y" and repeat up to +/-dy

Store results in {dx,dy} matrix
          sum { (green[x,y] - gmean) * (red[x,y] - rmean) }
Normalise based on variance

*/
```

The cross-correlation (CC_{G-R}) between the red and green channels was measured over a 9*9-pixel^2 region. The spatial registration of the two channels was systematically shifted in single-pixel steps over a +/- 4-pixel range (in both x and y) to find the highest Pearson's coefficient.

$$CC_{G-R} = \sum_{x=-4}^{4}\sum_{y=-4}^{4}(I_{Venus} - \overline{I_{Venus}})(I_{rhodamine} - \overline{I_{rhodamine}}) /\{ \left(\sum_{x=-4}^{4}\sum_{y=-4}^{4}\left(I_{Venus} - \overline{I_{Venus}}\right)^2\right)^{0.5} * \left(\sum_{x=-4}^{4}\sum_{y=-4}^{4}\left(I_{rhodamine} - \overline{I_{rhodamine}}\right)^2\right)^{0.5} \}$$

Similar operations computed $CC_{R\text{-}FRET}$ and $CC_{G\text{-}FRET}$.

c) Excel operations

1D array correlations (Pearson (P)) and statistical tests for significance (F-test, t-test) used the available Microsoft Excel functions.

APPENDIX-2: Performance of the step-finder algorithm

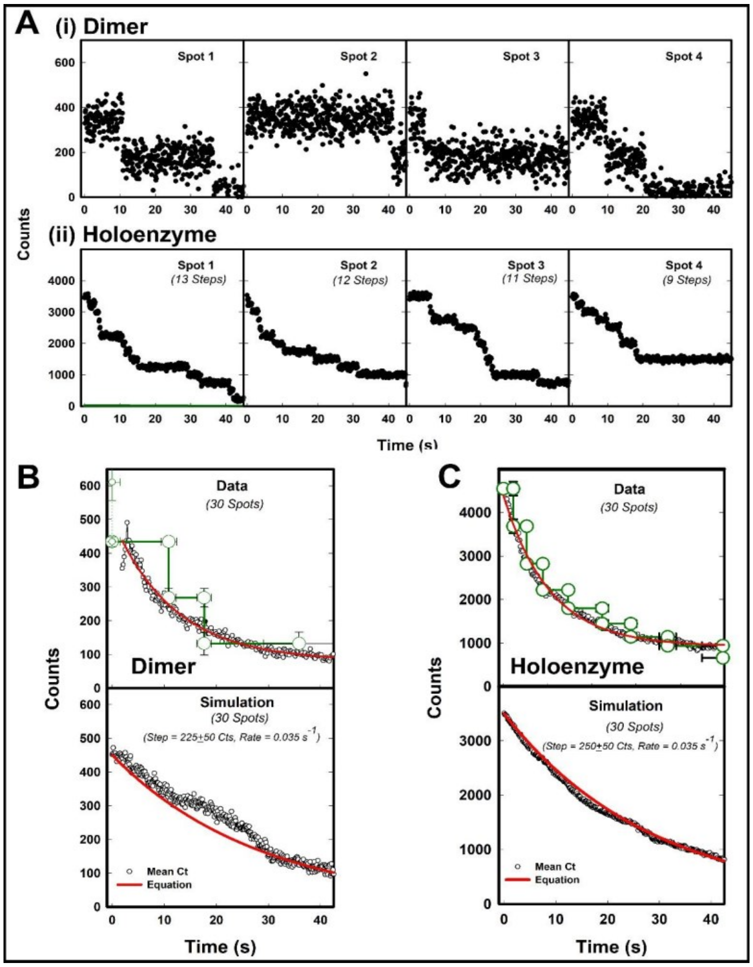

Figure A2.1. Simulation of photobleaching. A. *Examples of simulated single traces for* ***(i)*** *dimer and* ***(ii)*** *holoenzyme assemblies.* ***B.*** *Comparison of the average trace obtained from experimental versus simulated records of dimer assemblies.* ***C.*** *Comparison of the average trace obtained from experimental versus simulated records of holoenzyme assemblies. In* ***B,*** *and* ***C,*** *the observed noise and photobleaching decay exponent ($\pm$ 0.005 s^{-1}) in the experimental records was matched for the simulations. Red lines indicate both experimental fits and predicted decay. In addition to the averaged records (open circles), mean durations and amplitudes of experimental, sequential photobleaching steps are reported as stepwise drops in the mean step intensity change (green line). Green circles mark step start and end points ($\pm$ SE). The increased amplitudes of the early photobleaching events are probably due to residual errors in the subtraction of the autofluorescence background and missed short-duration steps that dominate the early record.*

The Microsoft Excel RAND() function was used to model the probability of stochastic photobleaching events, and the Gaussian-distributed noise comprising camera dark counts and photon noise as a function of spot intensity (I_0) and known EMCCD offset count value (usually ~100cts) using the transform.

$$Noise_{tot} = Offset + \left(\sqrt{I_0} + DarkNoise\right) * \sqrt{(-2 * LN(RAND()))} * COS(2\pi * RAND())$$

APPENDIX-3: The FRET signal.

The ALEX protocol was adapted from single-molecule FRET experiments (1). The amplitude of the FRET signal ordered by intensity was α-Dimer (300 > 30 nM) ~ β-monomer > β-dimer > β-holoenzyme (**Figure A3.1A**). Significant temporal correlations of the FRET signal with r-CAM colocalization were obtained for the β–monomer and dimer assemblies. The correlation for the β-dimer was worse than for the monomer. No measurable correlation was obtained for the β-holoenzyme. In all cases, the correlations were moderate. The Venus photobleaching drowned out anticipated anti-correlations between the Venus and FRET signals (**Figure A3.1B**).

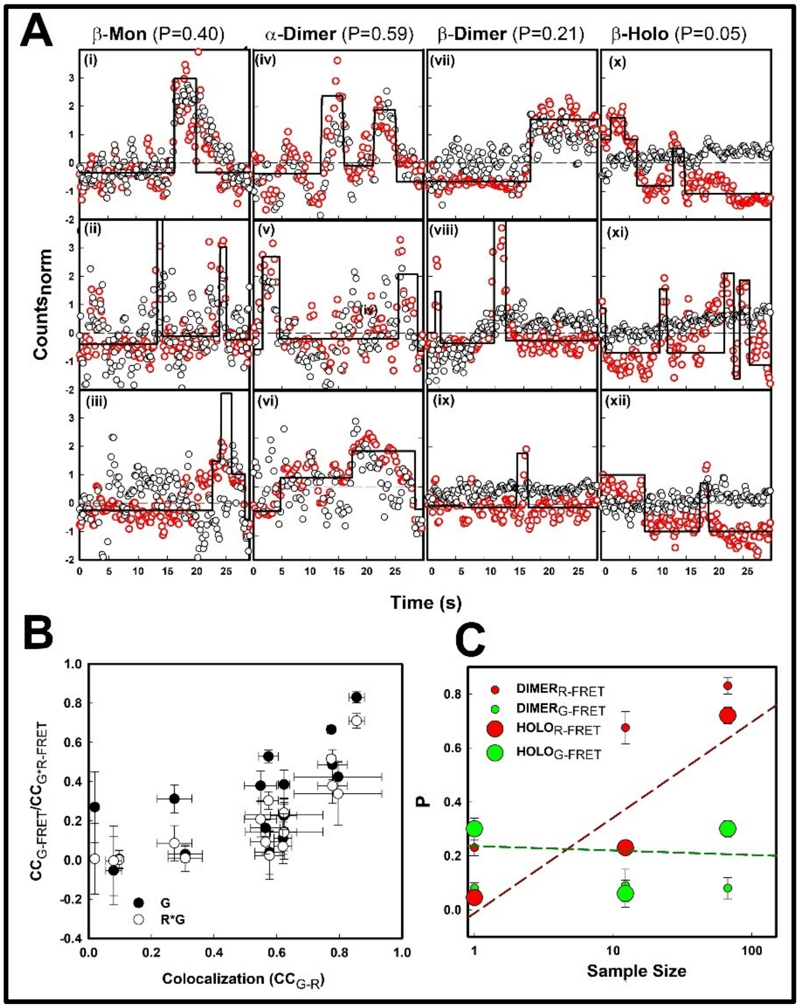

Figure A3.1: A. Temporal correlation between colocalization and FRET intensity. *Selected spot ROI records for the β monomer (**i-iii**), the α dimer (**iv-vi**), the β dimer (**vii-ix**), and β-holoenzyme (**x-xii**). Colocalization (red circles), FRET (light magenta circles). Steps reported by the step finder algorithm (black line).* ***B.*** *Correlations between the full-frame FRET versus G and G*R CCs with the colocalization (CC_{G-R}). The anti-correlation between the Venus and FRET signals is drowned out by Venus photobleaching The photobleaching dominates changes in the overlap integral, J.* ***C.*** *Simulations illustrate the improvement in the Pearson coefficient (P) for dimer and holoenzymes with sample size. The size ranged from single events (smallest) to a 10-spot image field (largest). The aggregate signal improved with size for the CC_{G-R} versus CC_{R-FRET}, but not the CC_{G-R} versus CC_{G-FRET} correlation. This is because the CC_{G-FRET} reflects Venus photobleaching, not N_{CAM} occupancy.*

The rankings can be rationalized based on the differences between linker lengths and fluorophore numbers that affect the FRET efficiency.

The FRET efficiency, $E_R = 1/\{1 + \left(\frac{r}{R_{50\%}}\right)^6\}$,

where r, the distance between the donor (Venus) and acceptor (rhodamine) within one subunit is 7±2 nm (Figure 2B). The $R_{50\%}$ is the Forster distance. The diffusion of the R peptide undocked from the KD core by bound r-CAM would supplement loop motions between the N and C-terminal KD lobes and between the tethered Venus and the N-terminal lobe to vary r for the monomer. In dimer and larger CaMKII assemblies, there will also be weaker energy transfer from Venus chromophores on adjacent subunits with the rhodamine. Their separation would vary (±4nm) depending on the fluctuations of the flexible KD-AD linkers. There is a stronger temporal correlation between the rhodamine and FRET channels for the α-dimer than the β-dimer consistent with the fact that the α-subunit has a shorter KD-AD linker (31 residues) than the β-subunit (93 residues). The fluctuations will also affect the excitation intensity profile along the z-axis of the evanescent field. The $R_{50\%}$ is 5.9 nm for the donor Venus and acceptor rhodamine-X pair. The $\left(\frac{r}{R_{50\%}}\right)$ ratio accounts for the weak FRET signals observed.

The simulation to study the dependence of the FRET signal on the Venus channel intensity fluctuations was based on the fluorescence energy transfer overlap integral, J.

The overlap integral, $J = C(R_{50\%}^6) = \int(E_D \, . A_A)\lambda^4 \, d\lambda$,

where E_D is donor emission, A_A is acceptor absorption. $C^{-1} = ((20.7/(128(\pi^5)N_A)) \times ((k^2)\mathrm{Q_D}/\mu^4)$ where N_A is Avogadro's number, $\mathrm{Q_D}$ is quantum efficiency and m is the dielectric constant. We observe acceptor emission but assume it is proportional to its absorbance of donor photons. Residual error in the subtraction of the leakage signal (10±1.5%) would contribute to the degradation of the CC_{G-FRET}.

APPENDIX-4: Power fits to the holoenzyme τ distributions.

We used the exponential fit equation (Figure 4B) to correct the photobleaching rate.

$$\int_{|\tau|=30}^{30} N_\tau/N_{\tau=0} = \left((1 - A_{PB})e^{-k_{\tau-corr}t}\right) + \left((A_{PB})e^{-\overline{N_{CAM}}.k_{PB}.t}\right)$$

This equation is accurate for the monomer, and an acceptable approximation for the dimer assemblies. The photobleaching correction was not applied to these distributions, in practice, as the short τ intervals meant that photobleaching kinetics had a minimal effect in practice on the monomer and dimer r-CAM distributions. It was applied to all holoenzyme distributions (Table 2). Single exponential fits were adequate. consistent with the limited spectral data range, for comparison of the holoenzyme (τ) distributions after photobleaching correction. A further correction is needed for the fact that k also depends on N_{CAM} occupancy. The modified equation is,

$$\int_{|\tau|=0}^{30} N_\tau/N_{\tau=0} = \left((1 - (A_{PB})_{\tau=0})e^{-\overline{N_{CAM}}.k_\tau.t}\right) + ((A_{PB})_{\tau=0}.\, e^{-\overline{N_{CAM}}.k_{PB}.t})$$

This correction was also not applied in practice as the N_{CAM} occupancy was similar between the inactive holoenzymes, while no information was obtained for the active T287D holoenzyme. The mean $\overline{N_{CAM}}$ occupancy is the average over the occupancy levels for all subunits in the holoenzyme. Therefore, a complete description would take the holoenzyme subunit stoichiometry into account.

$$\int_{|\tau|=0}^{30} N_\tau/N_{\tau=0} = (A)_\tau + (A_{PB})_\tau$$

approximates a power law where

$$(A_{PB})_\tau = \int_{S_{CaMKII=1}}^{14} \int_{|\tau|=0}^{30} ((A_{N_{CAM}^{S_{CaMKII}}})e^{-N_{CAM}^{S_{CaMKII}}.k_{PB}.t})$$

$$(A)_\tau = 1 - (A_{PB})_\tau = (\int_{N_{CAM=1}}^{14} \int_{|\tau|=0}^{30}((A_{N_{CAM}^{S_{CaMKII}}})e^{-N_{CAM}^{S_{CaMKII}}.k_\tau.t}))$$

We therefore compared exponential and power law fits to the βT287D $|\tau|$ distribution that approximated the holoenzyme photobleaching $|\tau|$ distribution at saturation N_{CAM} occupancy to better understand the complexity due to the multisubunit stoichiometry. The complete, normalized βT287D distribution is

$$(A_{PB})_{|\tau|<30} + (A_{PB})_{|\tau|>30} = 0.865 + 0.135 = 1$$

A three-exponential $((A_{PB})_{\tau-norm} = (0.1(e^{-0.2\tau}))+(0.053(e^{-0.01\tau})) + 0.1(e^{-0.07\tau}))$ fit (R=0.93) to the distribution is required to account for the $(A_{PB})_{\tau>30}$ fraction. As the number of exponents increases, multi-

exponential fits approximate a power law (2, 3). The three exponential fit is compared with a power law fit. While the fits to the < 30 s data are comparable (R=0.93), only the power law fit accounts for the > 30 s fraction (**Figure A2A**). The native and inactive holoenzyme distributions were corrected by subtraction of the inferred photobleaching events from the total events over t = 0->30s based on $(A_{kPB}{*}(e^{-k_{fPB}t}))$. The correction and single exponential fit (R=0.68) are illustrated for the native β holoenzyme (**Figure A2B**). The $k_{\tau 1-corr}$ distributions and fits for the inactive β holoenzymes (K43R (R=0.6), T287A (R=0.65), T287.306-307A (R=0.67)) are shown in **Figure A2C**. Their |τ| > 30s fractions have similar values to the βT287D fraction (0.14±0.02) since they are almost entirely due to the photobleaching of these multimeric assemblies.

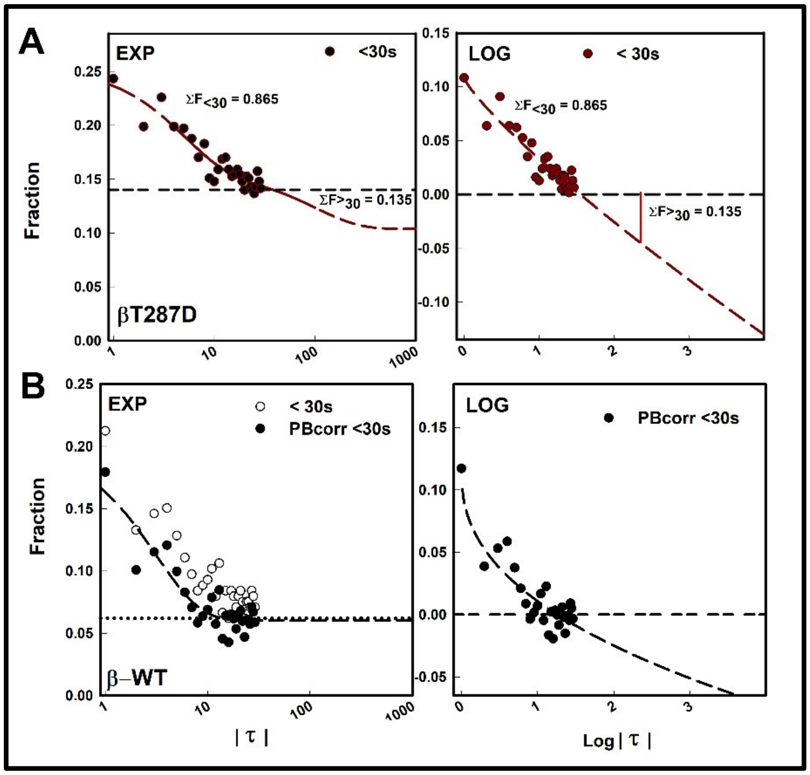

Figure A4.1: *Exponential versus power law fits for the active (βT287D) and inactive (βT287.306-307A) τ distributions.* ***A.*** *βT287D. Dashed red lines show the tri-exponential (EXP) and power (LOG) fits. Dashed black lines partition the τ <30s and >30s sub-populations. The τ <30s fraction is the area demarcated by the fit and the partition line. The τ >30s fraction is the area demarcated by the vertical red line (τ = 226s), the fit and the partition line. The tri-exponential fit will be extended well beyond τ = 226s to account for the | τ | >30s fraction.* ***B.*** *Native β. Comparison of the single exponential and power fits to the photobleaching-corrected (PBcorr) distribution.*

REFERENCES

1. Lerner, E., A. Barth, J. Hendrix, B. Ambrose, V. Birkedal, S. C. Blanchard, R. Borner, H. Sung Chung, T. Cordes, T. D. Craggs, A. A. Deniz, J. Diao, J. Fei, R. L. Gonzalez, I. V. Gopich, T. Ha, C. A. Hanke, G. Haran, N. S. Hatzakis, S. Hohng, S. C. Hong, T. Hugel, A. Ingargiola, C. Joo, A. N. Kapanidis, H. D. Kim, T. Laurence, N. K. Lee, T. H. Lee, E. A. Lemke, E. Margeat, J. Michaelis, X. Michalet, S. Myong, D. Nettels, T. O. Peulen, E. Ploetz, Y. Razvag, N. C. Robb, B. Schuler, H. Soleimaninejad, C. Tang, R. Vafabakhsh, D. C. Lamb, C. A. Seidel, and S. Weiss. 2021. FRET-based dynamic structural biology: Challenges, perspectives and an appeal for open-science practices. Elife 10.
2. Min, W., and X. S. Xie. 2006. Kramers model with a power-law friction kernel: dispersed kinetics and dynamic disorder of biochemical reactions. Phys Rev E Stat Nonlin Soft Matter Phys 73(1 Pt 1):010902.
3. Khan, S., I. Conte, T. Carter, K. U. Bayer, and J. E. Molloy. 2016. Multiple CaMKII Binding Modes to the Actin Cytoskeleton Revealed by Single-Molecule Imaging. Biophysical Journal 111(2):395-408.
