# Supplementary material for: Real-time single-molecule imaging of CaMKII-calmodulin interactions
Source: Biophys J. 2024 Feb 28;123(7):824–38. doi: 10.1016/j.bpj.2024.02.021 (PMC11630639; doi:10.1016/j.bpj.2024.02.021)
Supplement: Document S2. Article plus supporting material [file mmc2.pdf]

Biophysical Journal
Article

Biophysical Society

Real-time single-molecule imaging of CaMKII-calmodulin interactions

Shahid Khan,[1,*] Justin E. Molloy,[2] Henry Puhl,[3] Howard Schulman,[4,5] and Steven S. Vogel[3]
[1]Molecular Biology Consortium at Lawrence Berkeley National Laboratory, Berkeley, California; [2]The Francis Crick Institute, London, UK; [3]Laboratory of Biophotonics and Quantum Biology, National Institutes on Alcohol, Abuse and Alcoholism, National Institutes of Health, Rockville, Maryland; [4]Panorama Research Institute, Sunnyvale, California; and [5]Stanford University School of Medicine, Stanford, California

ABSTRACT The binding of calcium/calmodulin (CAM) to calcium/calmodulin-dependent protein kinase II (CaMKII) initiates an ATP-driven cascade that triggers CaMKII autophosphorylation. The autophosphorylation in turn increases the CaMKII affinity for CAM. Here, we studied the ATP dependence of CAM association with the actin-binding CaMKIIβ isoform using single-molecule total internal reflection fluorescence microscopy. Rhodamine-CAM associations/dissociations to surface-immobilized Venus-CaMKIIβ were resolved with 0.5 s resolution from video records, batch-processed with a custom algorithm. CAM occupancy was determined simultaneously with spot-photobleaching measurement of CaMKII holoenzyme stoichiometry. We show the ATP-dependent increase of the CAM association requires dimer formation for both the α and β isoforms. The study of mutant β holoenzymes revealed that the ATP-dependent increase in CAM affinity results in two distinct states. The phosphorylation-defective (T287.306-307A) holoenzyme resides only in the low-affinity state. CAM association is further reduced in the T287A holoenzyme relative to T287.306-307A. In the absence of ATP, the affinity of CAM for the T287.306-307A mutant and the wild-type monomer are comparable. The affinity of the ATP-binding impaired (K43R) mutant is even weaker. In ATP, the K43R holoenzyme resides in the low-affinity state. The phosphomimetic mutant (T287D) resides only in a 1000-fold higher-affinity state, with mean CAM occupancy of more than half of the 14-mer holoenzyme stoichiometry in picomolar CAM. ATP promotes T287D holoenzyme disassembly but does not elevate CAM occupancy. Single Poisson distributions characterized the ATP-dependent CAM occupancy of mutant holoenzymes. In contrast, the CAM occupancy of the wild-type population had a two-state distribution with both low- and high-affinity states represented. The low-affinity state was the dominant state, a result different from published in vitro assays. Differences in assay conditions can alter the balance between activating and inhibitory autophosphorylation. Bound ATP could be sufficient for CaMKII structural function, while antagonistic autophosphorylations may tune CaMKII kinase-regulated action-potential frequency decoding in vivo.

SIGNIFICANCE The calcium/calmodulin-dependent protein kinase II (CaMKII) is an intricate molecular machine that uses ATP to phosphorylate itself as well as substrates once activated by the second messenger calcium-calmodulin (CAM). ATP regulation of CAM affinity is critical to CaMKII function in excitable cells. We analyzed the CAM occupancy and residence time distributions of isolated native, and mutant CaMKII populations molecule-by-molecule with a time-resolved, high-throughput, two-color fluorescence assay. We find that ATP switches CaMKII between distinct CAM affinity states determined by CaMKII domain architecture, oligomerization, and the balance between inhibitory and activating autophosphorylations. CAM molecules bind CaMKII holoenzyme subunits independently of each other with characteristic state-dependent probabilities. Our observations constrain how CaMKII might decode calcium pulse frequencies.

INTRODUCTION

Calcium/calmodulin (CAM) is a ubiquitous second messenger in eukaryotic signal transduction circuits. Upon binding calcium, the protein undergoes large conformational changes (1). Target proteins have evolved a distinct CAM binding motif (2). The fast calcium loading/unloading kinetics make the subsequent

Submitted August 3, 2023, and accepted for publication February 22, 2024.

*Correspondence: smkhan@lbl.gov

Justin E. Molloy's present address is CMCB, Warwick Medical School, Coventry CV4 7AL, UK

Editor: Dylan Myers Owen.

https://doi.org/10.1016/j.bpj.2024.02.021

© 2024 Biophysical Society.

This is an open access article under the CC BY license (http://creativecommons.org/licenses/by/4.0/).

CAM/target interaction the rate-limiting step in CAM-mediated signal relays (3).

The calcium/calmodulin-dependent protein kinase II (CaMKII) is an important CAM target because CaMKII phosphorylates multiple substrates to initiate Ca^{2+} signaling cascades (4). Remarkably, CaMKII holoenzymes act as individual, action potential frequency decoders by tuning kinase activation to calcium pulses (5). Frequency decoding may be central to CaMKII physiology in excitable cells. CaMKII architecture (Fig. 1) has a common design across species—a central hub assembled as dimer pairs from a conserved association domain (AD), separated by a linker from an equally conserved kinase domain (KD) (6). The KDs radiate out from the AD hub (six pairs/dodecamer, seven pairs/tetradecamer (7)). The KD has a canonical fold, with the ATP binding pocket between the N- and C-terminal lobes (8). CAM binds to the KD regulatory domain (R), undocking it from the substrate binding cleft in the autoinhibited state (9,10). The linker has variable length and sequence between mammalian isoforms (α, β, γ, δ) and developmental splice variants (11). The

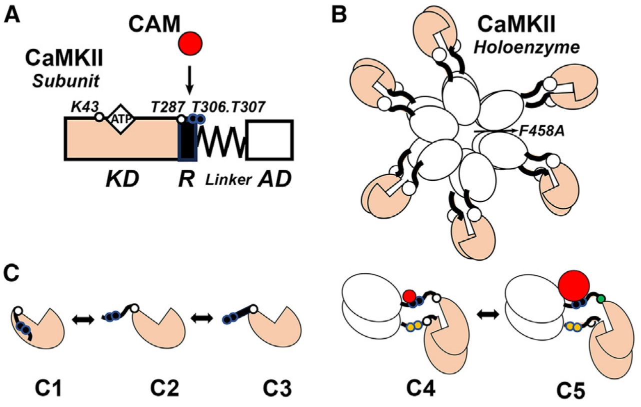

FIGURE 1 CaMKII architecture and dynamics. (A) CaMKII subunit. The N-terminal canonical KD and the C-terminal AD are connected via a flexible linker whose length and composition vary between isoforms and their splice variants. Residue substitutions at the KD K43, T287, T306.T307 (β isoform residue positions) are examined in this study. K → R residue substitution at K43 (*open circle*) disrupts the ATP binding site (*diamond*). The regulatory autoinhibition segment (R) binds CAM (*red circle*) and contains the activating (T287, *open circle*) and inhibitory (T306.T307, *closed circles*) autophosphorylation sites. T → A residue substitutions silence autophosphorylation, while T → D substitutions mimic it. (*B*) CaMKII holoenzyme (dodecamer). The AD forms a central 2-stack hub with mirror symmetry. The peripheral KDs from the two stacks are predominantly paired. The AD residue substitutions βF458A, αF394A destabilize lateral hub contacts. (*C*) CaMKII conformational states. R conformational heterogeneity reported by ESR for the monomeric KD consists of at least three states; a docked state (C1) and unstructured C2), or α-helical, CAM-bound (C3) undocked states (23). Dimer formation pairs catalytic KDs, as seen by Homo-FRET (C4) (54). Experiments with R peptides show T287 transphosphorylation (*green circle*) accesses a higher affinity (*depicted as large red circle*) "trapped" CAM state (C5). Catalytic pairs are maintained in dimers or holoenzymes in the presence of CAM or phosphorylated T287 (17). To see this figure in color, go online.

α and, to a lesser extent, β isoforms are predominantly expressed in the brain, while the γ and δ isoforms are broadly distributed (12).

The mechanism of CAM-triggered CaMKII kinase activation, based mostly on the α isoform, has been studied with fluorophore-tagged CAM binding assays using population anisotropy (13) or quenching (10,14–16) complemented with kinase assays (5,17,18), in vivo fluorescence energy transfer (FRET) (19), and structural data (6,20). AD mutations, such as α F394A, abolish lateral contacts to disassemble the holoenzyme into six dimers (18). In dimers and higher-order assemblies, the undocked R of one activated subunit is captured by an adjacent subunit substrate binding site freed of its own R by CAM binding for transphosphorylation of the captured R residue T287 (20). Here and throughout, the residue numbering is based on the β isoform. The intraholoenzyme transautophosphorylation allows CaMKII concentration-independent kinase activation (21). At the high CaMKII concentrations in dendritic spines in CNS neurons, interholoenzyme trans-autophosphorylation can also occur (22). Distinct conformational states based on ESR of R dynamics (23) or FRET of catalytic pairs (18,24) have also been identified in addition to the kinase activation states (Fig. 1). The multimeric ring architecture of CaMKII, unique among the ~80-member CAM kinase superfamily (4,25), has intrinsic polymorphism and lability to add to the conformational heterogeneity (7).

In previous work, we applied time-resolved, dual-color total internal reflection fluorescence microscopy (TIRFM) to report that the dominant actin binding CaMKIIβ isoform (26) forms resilient, multivalent actin networks that are rapidly disassembled by CAM for remodeling by myosin motors (27,28). Here, we use TIRFM for measurements of the association/dissociations of rhodamine-labeled CAM molecules from single, immobilized, Venus-tagged CaMKII holoenzymes. The rhodamine/Venus colocalization allowed study of the kinetic complexity of the CAM/CaMKII association, while stepwise Venus photobleaching revealed the stoichiometry of the CaMKII assembly. TIRFM of single holoenzymes eliminated interholoenzyme reactions (22) and subsequent video analysis separated them from smaller CaMKII complexes generated by holoenzyme disassembly (7).

ATP has diverse effects on CaMKII function and physiology (4). 1) ATP is, of course, essential for the phosphorylation of multiple substrates to regulate a broad range of signal transduction circuits (29). 2) It is also used for multiple autophosphorylations of CaMKIIα; T286 autophosphorylation results in autonomous activation, while αT305.306 autophosphorylation inhibits CAM reassociation (21). Autophosphorylation of S331 and S371 in the linker region regulates actin binding in the β isoform (4). 3) Bound ATP stabilizes the human CaMKIIα KD (30). Occupancy of the ATP binding site by a nucleotide, even an inhibitor

(AS283), is required to facilitate displacement of the R domain and binding of the NMDA receptor, an essential step for long-term potentiation (LTP) and synaptic memory (31). 4) Molecular simulations of the CaMKII KD dynamic network show that the ATP binding pocket is the network node as in canonical kinases like protein kinase A (32), but now coupled to both the substrate and CAM binding sites. Bound ATP closes the hinge between the N- and C-lobes to constrain lobe flexibility, while intersubunit capture further freezes the R1 segment to influence long-range couplings (8). Bound nucleotide and substrate have reciprocal effects on their respective affinities (33–36), in line with the simulations.

Remarkably, the ATP-triggered αT286 autophosphorylation causes a 1000×-fold increase in CAM affinity (13). However, ATP also increases CAM binding 9-fold to the mutant αT286A that cannot undergo autophosphorylation (37). Here, we aimed to disentangle the autophosphorylation-dependent and -independent effects, given that LTP does not require autophosphorylation (31). We studied the CAM/CaMKII association with and without ATP as a function of isoform, assembly state, and autophosphorylation with a panel of site-specific residue substitutions. These substitutions disassemble the holoenzyme and target the ATP binding and autophosphorylation sites as indicated (Fig. 1). We found that ATP generates two distinct CaMKII affinity states for CAM. The low-affinity state requires dimerization but not autophosphorylation. The high-affinity state requires T287D, a mimic of T287 autophosphorylation, and matches the previously reported trapped CAM PhosphoT287-CaMKII state (13). The holoenzyme CAM occupancy follows the Poisson distribution in both low- and high-affinity states.

MATERIALS AND METHODS

Protein expression and purification

Reagents were sourced from Sigma-Aldrich (Gillingham, Dorset, UK), except for wheat germ rhodamine-calmodulin (Stratech Scientific, Ely, Cambridgeshire, UK); mouse monoclonal GFP antibody no. 1814460 (Roche, Basel, Switzerland) and GFP protein, no. 8365-1 (Clontech, Mountain View, CA). Free rhodamine in the rhodamine-calmodulin stock, if any, was removed by a Sephadex G-25 desalting column. The bleached stock fraction was <20% as assessed by comparison of the protein (A280) and rhodamine absorbance (A550). The Stratech Scientific rhodamine calmodulin conjugated rhodamine-X maleimide with the wheat germ calmodulin single cysteine residue. The product has been discontinued, but rhodamine-X maleimide remains available (Lumiprobe, Hunt Valley, MD) together with wheat germ calmodulin expression plasmids (38). The mouse Venus-CaMKII (V-CaMKII) fusion constructs for mammalian cell expression were adapted for His-tagged expression in bacterial hosts using the plasmid pSMT3 as the expression vector (18,28). Table S1 details the construction of the bacterial expression plasmids and functional phenotypes of the mutant CaMKIIs. The parent strains were competent for primary autophosphorylation (7).

The plasmids were cotransformed with a plasmid encoding protein phosphatase into BL21/Rosetta or BL21/C41. Single colonies with high-yield expressions were selected. Proteins were purified from 1 L cultures, inoculated from the colony stocks, by nickel affinity chromatography; with the peak fractions pooled and concentrated with 300 kDa MW cutoff centrifugal filters (Amicon Ultracel), all as described (7). The concentrated peak fractions were flash-frozen and stored at −80°C.

The V-CaMKII and rhodamine-CAM stocks were thawed (4°C) then diluted into a degassed AB^- buffer (25 mM imidazole-HCl, 25 mM KCl, 1 mM EGTA, 4 mM $MgCl_2$ [pH 7.4]) immediately before the experiment. Desired rhodamine-CAM concentrations were obtained by serial dilution from the 55.5 mM stock. The AB^- was supplemented with 0.5 mg/mL bovine serum albumin (BSA) (AB^-.BSA), 0.5 mM calcium in EGTA-free AB^- without ($AB^-.Ca^{2+}$) or with ($AB^+.Ca^{2+}$) 2 mM ATP as needed. Rhodamine-CAM loaded with Ca^{2+} will be henceforth referred to as r-CAM. The buffers were supplemented with oxygen scavenger mix (0.2 mg/mL glucose oxidase, 0.5 mg/mL catalase, 0.5 mg/mL BSA, 3 mg/mL glucose) in HPLC-grade distilled water before the microscopy (28).

Microscopy

The dual-camera TIRFM microscope workstation is illustrated in Fig. 2 *A*. The design is an advance from the single-camera workstation used earlier (27). The sample was imaged with alternate blue/olive laser excitation. The camera images were acquired at 48 frames/s and overlaid with a modified version of GMimPro (39). The effective magnification was 100 nm/pixel (512 × 512 pixels/frame). A flow-chamber was assembled from two glass coverslips joined with tape and fixed in a matching slot on the microscope stage. The coverslip surface was sparsely populated with GFP antibody before adsorption was blocked by AB^-.BSA. The V-CaMKII sample was then flowed in and incubated for 15 min. Unbound V-CaMKII was washed out and r-CAM at the desired concentration in $AB^-.Ca^{2+}$ or $AB^+.Ca^{2+}$ washed in (Fig. 2 *B*). The recordings were started concurrently or just before the laser shutters were opened, and the stage moved immediately to an unilluminated, adjacent area once the TIRF image was brought into focus. This protocol ensured that the initial photobleaching events from holoenzyme assemblies were not missed.

Video analysis

The "green" and "red" EMCCD cameras were electronically synchronized, and video data recorded at 100 ms per frame (∼98 ms laser exposure time) were saved as two separate movie files (∼500 frames). The movies were saved in a custom binary format consisting of 16-bit raw camera pixel data with a short metadata header for every video frame. Video frames were interleaved because the alternating laser excitation was triggered by the camera synchronization pulses. The movie stacks were analyzed using a combination of standard digital image processing algorithms, automated using the ImageJ (version 1.53f) macro language to quantify the CaMKII stoichiometry and CAM occupancy. The analysis pipeline is described below.

1) *Registration:* the two movie files were loaded into computer memory and spatially registered using the built-in ImageJ rotation and translation operations with parameters previously optimized with images of fixed, multiwavelength emission, TetraSpeck microspheres (Thermo Fisher Scientific, Loughborough, Leicestershire, UK).
2) *Deinterleave:* movies were deinterleaved to yield four channels ("stacks" in ImageJ) corresponding to the two fluorescence emission channels from the green and red cameras recorded during the blue/olive alternating laser excitation. The "Venus channel" is the green camera record of the V-CaMKII fluorescence excited by the blue laser. The camera records no fluorescence during the olive laser excitation and this blank record is discarded. The "rhodamine channel" is the red camera

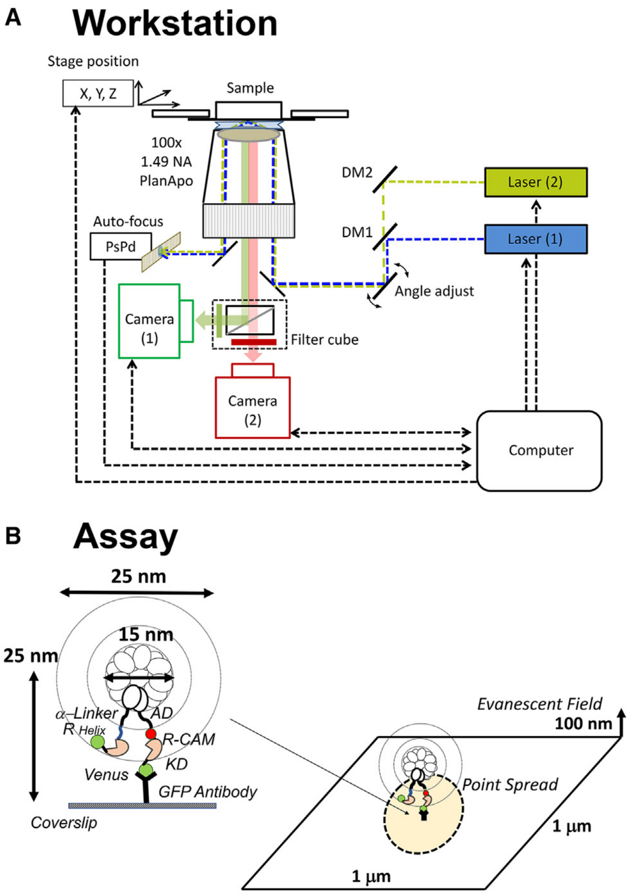

FIGURE 2 (*A*) Workstation. The workstation recorded dual color fluorescence emission from alternate excitation by two lasers that was filtered and imaged by two cameras. The lasers (*blue*, 488 nm [laser 1]; *olive*, 561 nm [laser 2]) (Lighthub-6, Omicron, Rodgau-Dudenhofen, Germany) and cameras (iXON-3, EMCCD, Andor, Belfast, Northern Ireland) are computer controlled. The filter cube had green (525 ± 50 nm) and red (593 ± 40 nm) barrier filters with dichroic (Di02-R561) (Semrock, Laser2000, Huntingdon, Cambridgeshire, UK). Cameras 1 and 2 were designated green and red, respectively, based on the associated barrier filters. The specimen was illuminated by an Olympus TIRF UPLAPO100xOHR, 1.5NA objective lens (Olympus, Southend-on-Sea, Essex, UK). The custom-built autofocus mechanism used a position-sensitive photodiode to measure the deflection of the returning, reflected laser beam (PSD S1352, Hamamatsu Photonics, Welwyn Garden City, Hertfordshire, UK). The field of view and total internal reflection angle were set by manual adjustment of a custom beam expander, field diaphragm, and kinematic mirror, which for clarity are shown as a single adjustable mirror. All components were assembled on a custom-built frame (Thorlabs, Ely, Cambridgeshire, UK). (*B*) TIRFM Assay. The microscope flow cell, consisting of two coverslips, held together by parallel strips of double-sided adhesive tape, was mounted on a three-axis piezo stage (XYZ-SLC17:22 with MCS-3c, SmartAct, Oldenburg, Germany). One surface was sparsely coated with GFP antibody and then blocked with BSA. V-CaMKII incubation was optimized to minimize overlap of the point spread functions with mean separation of the bound V-CaMKII >2 mm. The centroid of the bound molecules was one-quarter of the decay distance of the exponential field. The CAM binding motif is located on the R segment 7 ± 2 nm away from the nearest and 22 nm away from the furthest Venus fluorophores in the holoenzyme. To see this figure in color, go online.

record of the r-CAM emission excited by the olive laser. During blue laser excitation, the red camera records a mixed signal composed of leakage of the Venus fluorescence through its barrier filter, and FRET from Venus to adjacent rhodamine fluorophores.

3) *Leakage correction:* the leakage was estimated from records of V-CaMKII holoenzymes in the absence of r-CAM. It is 10 ± 1.5% of the Venus channel intensity. The leakage was corrected by subtraction of the corresponding fraction of the latter intensity from the mixed channel. The residual intensity after leakage correction is the FRET channel.
4) *Uneven field correction:* the uneven illumination was corrected using the built-in ImageJ rolling-ball background filter (100-pixel ($\pi \times (10\ \mu m)^2$) radius) to remove low spatial frequency intensity variation from the average image.
5) *ROI identification:* spots corresponding to individual r-CAM and V-CaMKII molecules were identified in the rhodamine and Venus channels, respectively, based on their diffraction-limited spot size and integral intensity. Movie stacks were z-projected to yield a single averaged frame. The morphological filter used comprised a 7×7 pixel^2 kernel that approximates a "Laplacian of a Gaussian" function and an intensity thresholding algorithm. The center of each spot was the center of a 5×5 pixel^2 ($\sim 500 \times 500$ nm^2) region of interest (ROI) that encompassed >80% of the spot intensity.
6) *Autofluorescence correction:* autofluorescence due to microscope coverslip surface contamination, excited mostly by the blue laser, was measured as the integrated frame intensity with the ROIs excluded. The fluorescence decay for each channel was fitted to a dual-exponential with a constant offset. This function was then subtracted from all pixels, including ROI pixels, per frame with the ImageJ "math macro function" over the time course of the records.
7) *Global cross correlation:* a global measure of correlation was obtained from the projected images (512×512 pixel^2, 32-bit) of the mean intensities for the Venus and rhodamine channels. The correlation coefficient (CC) was sensitive to the image registration, so the rhodamine image was translated relative to the Venus image +/− 4 pixels relative to the original image registration in single-pixel steps. The 9×9 matrix of CCs is shown (Fig. 3*Ai*). The peak CC value was used to measure the colocalization ($CC_{G\text{-}R}$). Similar operations were performed to measure the cross correlations of the FRET channel with the Venus ($CC_{G\text{-}FRET}$) and rhodamine ($CC_{R\text{-}FRET}$) channels.
8) *Simulations:* single molecule data sets are noisy due to shot noise from the weak fluorophore signals, camera noise, and background autofluorescence; further compounded by the stochastic nature of single-molecule interactions. So, virtual movies were generated and simulated with realistic noise levels and temporal fluorophore behaviors to assess various aspects of our analysis pipeline, including the comparison of the observed CCs with CCs obtained from virtual image fields with set numbers of V-CaMKII, r-CAM and their complexes (Fig. 3 *Aii*).
9) *Step finding algorithm:* each spot ROI was assigned a unique identifier ID. The spot IDs, locations, and intensity versus time profiles were saved as comma-delimited format, ASCII files for off-line analysis. Another ImageJ macro detected fluorescence step events for each spot in the time record using a four-pass custom step-finder algorithm. Parameters used in each pass of the algorithm could be adjusted interactively (using "sliders") and superposition of the modeled stepwise intensity changes plotted over the raw intensity versus time data gave a visual check of data fitting and residuals. The first derivative of the spot intensity was computed over a rolling window of size "a" after the removal of high-frequency fluctuations by a sliding window of "b" points. Prominent peaks in the output identified abrupt intensity changes using a quality index "q" threshold (ImageJ "Analyze Peaks" function). Peaks with intensity changes "ΔI" within one standard deviation of the mean intensity of the monomeric Venus-tagged KD step distribution were reported as steps. Interactive sliders optimized a, b, and ΔI slider options for the spot population from a few (<5) spot records. The critical options were the duration (>0.3 s) of stationary intensity levels and the amplitude of

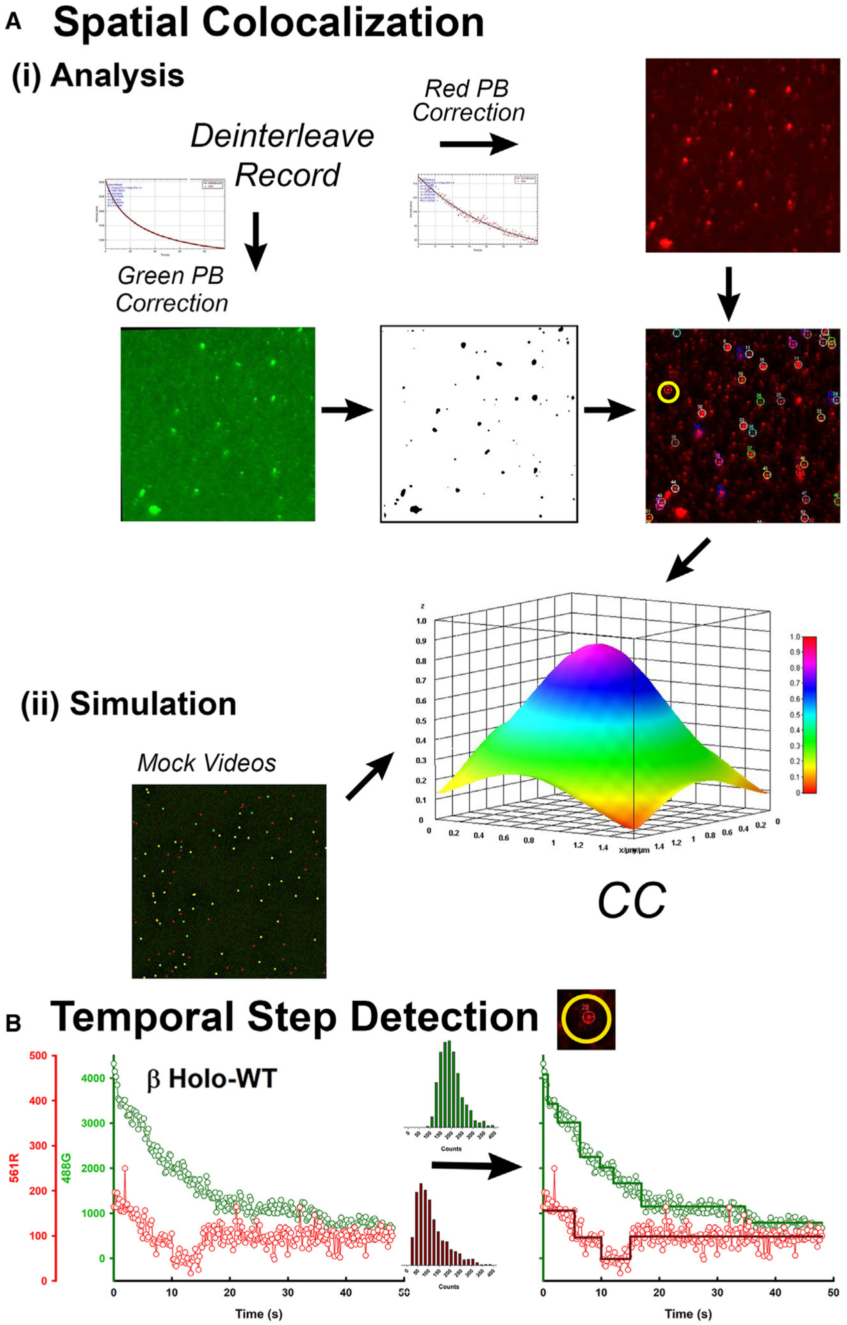

FIGURE 3 (*A*) Spatial cross correlation. (*i*) Analysis.: alternate laser excitation separated red and green fluorophore emissions and FRET between green and red fluorophores. Rhodamine emission (r-CAM) was measured by the “red” camera during 561 nm laser excitation. Venus emission (V-CaMKII) was measured by the “green” camera during 488 nm laser excitation. Cross correlation “CC” between the Venus and rhodamine channels was computed for a moving 9 × 9 pixel^2 region. PB, photobleaching. (*ii*) Simulation: mock vidoes were processed similarly. (*B*) Temporal step detection. Left panel: colocalized rhodamine (CAM, *red circles*) and Venus (CaMKII, *green circles*) intensity versus time data from a single spot ROI (*yellow circle* in *Ai*). Right panel: the fits to the Venus and rhodamine channels to a stepwise fluctuation model. The trajectories were filtered using a three-frame running average. The immobilized V-CaMKII shows a monotonic intensity decay due to photobleaching, whereas the r-CAM trajectory shows step increases and decreases. Characterization of an intensity change as a step event was based on the mean (± SD) values for the intensity distributions shown for the monomeric-tagged KD (*green*) and the nonspecifically attached r-CAM (*red*). To see this figure in color, go online.

the intensity jumps between levels. The steps were due to photobleaching or changes in the occupancy of r-CAM molecules (N_{CAM}) colocalized with the V-CaMKII. Stepwise photobleaching events in the Venus channel gave an estimate of CaMKII subunit stoichiometry (S_{CaMKII}). The jumps expected from single fluorophore photobleaching were determined from the measurement of monomeric V-CaMKII and nonspecifically attached r-CAM intensity distributions (Fig. 3 *B*). The output saved as ASCII files, as for the Spot Finder ImageJ macro, could be analyzed with Student’s *t*-test (unequal variance option) to score the significance of the intensity differences between levels.

Observed V-CaMKII step intensity distributions matched expectations as reported elsewhere (7). Additional simulations checked that the photobleaching decay averaged over a virtual spot population of size typically obtained in our experimental image fields matched the full-frame photobleaching decay. Analysis of temporal intensity fluctuations due to association/dissociation of single r-CAMs in the rhodamine channel was similar to the Venus photobleaching analysis, except that parameters were now optimized for the rhodamine fluorophore.

A key to the abbreviations used and the operations are given in Appendix-1. The simulation to evaluate the performance of the step-finder algorithm are described in Appendix-2. The analysis of the FRET signal is detailed in Appendix-3. All ImageJ macros and simulations are available on GitHub. Graphical plots. Fits were made with Sigmaplot version 12 (Systat Software, San Jose. CA).

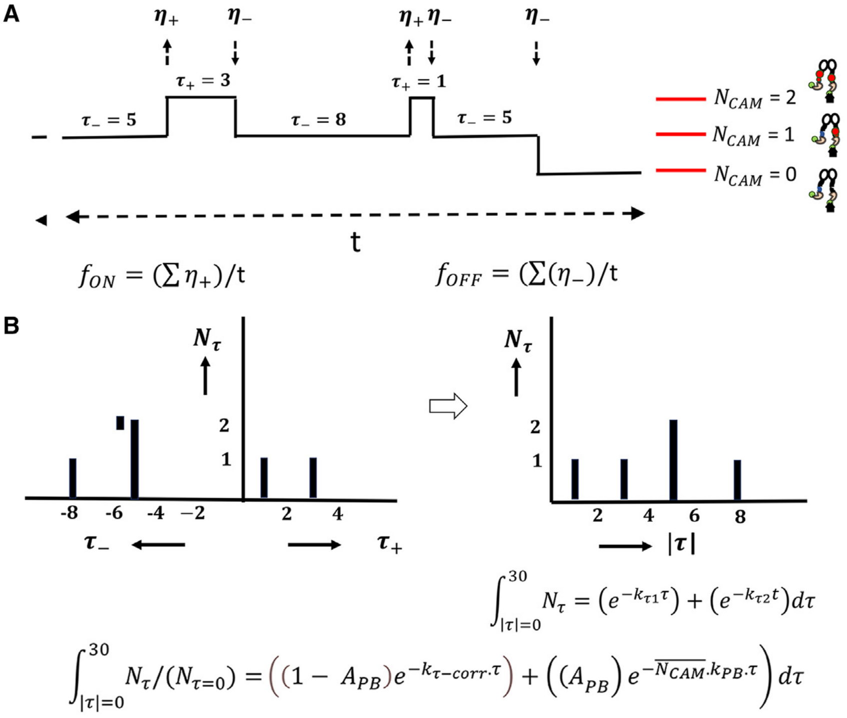

FIGURE 4 Schematic. An idealized rhodamine channel "red" record with stepwise r-CAM associations/dissociations from an immobilized V-CaMKII ("green") dimer spot. The schematic illustrates how f_{ON}, f_{OFF} frequencies and k_τ rates were computed. (*A*) The f_{ON},f_{OFF} frequencies were obtained from the number of up and down step events, respectively. (*B*) The k_τ values were obtained from single or double exponential fits to the residence time (τ) distribution over all nonzero occupancy levels. N_τ = number of intervals per |t| value. The k_τ ($= k_{\tau 1}$ for biexponential decay) could be compared with, or corrected for, the photobleaching rate (k_{PB}) and amplitude (A_{PB}). $k_{\tau-corr}$ denotes k_τ corrected for the photobleaching decay. The records for the monomer and dimer assemblies were not corrected. To see this figure in color, go online.

Kinetic analysis

Steps were scored in the rhodamine channel record when the amplitude of the intensity jump was within one standard deviation of the mean of the nonspecifically attached r-CAM distribution. The frequency of OFF events, f_{OFF}, was calculated from the number of stepwise intensity decreases in a single spot record divided by the record duration The frequency of ON events, f_{ON}, was calculated from the number of stepwise intensity increases in a single spot record divided by the record duration. (Fig. 4 *A*). The f_{OFF} does not distinguish between intensity decreases due to dissociation versus those due to photobleaching. The f_{ON} is related to, but different from, the actual association rate as it does not account for the abundances of the reactants—the r-CAM concentration and the CaMKII subunit stoichiometry—that determine the order of the reaction. Neither metric takes the N_{CAM} occupancy level into account.

The k_τ values were computed from exponential fits to the residence time $|(\tau)|$ distributions obtained over all nonzero N_{CAM} occupancy levels (Fig. 4 *B*). The distributions were limited to τ values $< \pm 30$ s. The fractions contributed by the $|\tau| > 30$ s subpopulations were recorded, but their τ distributions were not analyzed as they are dominated by photobleaching. As with f_{ON} and f_{OFF}, the k_τ values are approximations that ignore the τ dependence on N_{CAM} occupancy. However, they have the advantage that they can be compared with, and corrected for, the photobleaching rate. The k_τ values were primarily used as the default apparent dissociation rate for comparison of the various V-CaMKII constructs, even though both k_τ and f_{OFF} values are tabulated.

In our assay, steady-state N_{CAM} occupancy is reached in the dark before laser excitation. Net flux is zero at steady state (f_{ON} due to association = f_{OFF} due to dissociation). In contrast to the actual occupancy, the observed N_{CAM} occupancy recorded upon laser excitation will decrease. In addition to dissociation, there will be additional (n_-) events due to photobleaching. The ($\sum n_+ / \sum n_-$) ratio is a useful metric for an initial assessment of the photobleaching contribution. The $|\tau|$ intervals are set by two probability distributions, for association/dissociation and photobleaching. We define an affinity index, A_i = {r-CAM(k_τ/f_{ON})}, a heuristic substitute for the dissociation constant (K_D), which allows comparison of the relative affinities of the different assemblies and mutants. Fits to the holoenzyme τ distributions that consider the dependence of τ on N_{CAM} occupancy level including the $|\tau| > 30$s fractions are shown in Appendix-4.

RESULTS AND DISCUSSION

CAM binding kinetics in β holoenzymes and subassemblies

ATP regulation of r-CAM association requires dimer formation

The single-spot Venus intensity distributions for the different V-CaMKII assemblies were converted into subunit stoichiometry distributions by processing spot photobleaching records with a custom step-finder algorithm (7). Frame-by-frame CCs ($CC_{G\text{-}R}$) measured V-CaMKII/r-CAM colocalization over the complete data set for each experimental condition (Fig. 3 *A*). The colocalization for single V-CaMKII spots as a measure of bound r-CAM was supported by parallel measurements of cross correlations between the rhodamine/FRET ($CC_{R\text{-}FRET}$) and Venus/FRET ($CC_{G\text{-}FRET}$) channels.

Calcium (Ca^{2+}) was indispensable for colocalization. The ($CC_{G\text{-}R}$) in the absence of Ca^{2+} was <0.03 regardless of r-CAM concentration, ATP or V-CaMKII assembly state. The Venus-tagged KD ($\beta_{\Delta 315}$), will henceforth be cited as the "monomer." Its $CC_{G\text{-}R}$ values at 300 nM CAM were similar in the presence and absence of 2 mM ATP. The Venus-tagged AD mutants (α_{F394A}, β_{F458A}) will henceforth be cited as "dimers." The ($CC_{G\text{-}R}$) values of the dimers

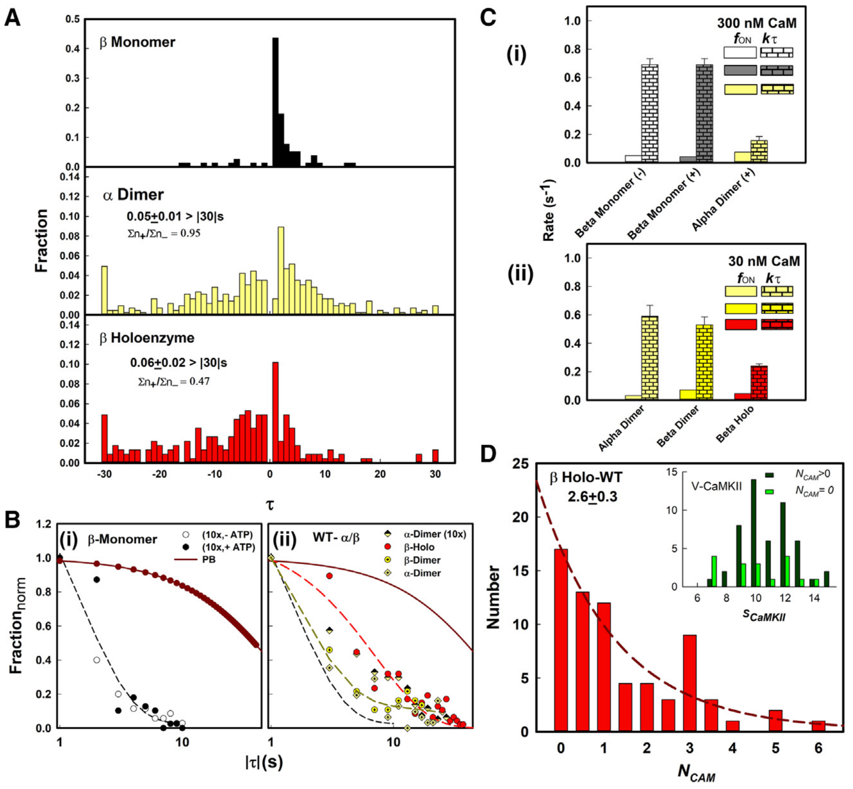

FIGURE 5 Association and dissociation kinetics of native assemblies. (*A*) Histograms. Residence times (τ) over the −30 to +30 s range (2 mM ATP). For each species, 20–30 spots were selected at random from >2 different records from separate experiments. The step population (Σn) = 175 (β monomer), 555 (α dimer), and 226 (β holoenzyme). Record duration = 40 s. (*B*) Residence time ($|\tau|$) distributions. (*i*) The β monomer distributions. Single exponential fit (*short dashed line*). Monomer r-CAM photobleaching (PB) curve (*red line*). (*ii*) The α dimer, β dimer, and β holoenzyme (t) distributions. Double exponential fit (α dimer, *black dashed line*). Single exponential fit (β holoenzyme, *red dashed line*). The PB curve and β monomer fit are as in (*i*). (*C*) Rates. (*i*) Mean (f_{ON}) and (k_τ) at 300 nM r-CAM for the β monomer (+/− ATP) and α dimer. (*ii*) Mean (f_{ON}) and (k_τ) at 30 nM r-CAM for the α dimer, β dimer, and β holoenzyme. f_{ON} (*clear*), k_τ (*brick*). (*D*) The r-CAM occupancy distribution (30 nM r-CAM) for the β holoenzyme. Single exponential fit (*red dashed line*). Inset: the means of the V-CaMKII subunit stoichiometry (S_{CaMKII}) distributions obtained from PB analysis were 10 ± 2 and 10.5 + 1.5 s for zero (0) and nonzero (>0) subpopulations, respectively. The F-test probability, P_{F-test} = 0.0035, indicated unequal variance. The *t*-test probability with the unequal variance option, P_{t-test} = 0.085 did not show a significant difference between the mean values. To see this figure in color, go online.

and holoenzyme (β) constructs depended on ATP. In 2 mM ATP, higher $CC_{G\text{-}R}$ values were obtained for the holoenzyme at an order of magnitude lower r-CAM concentration relative to the dimer constructs. Computer simulations related $CC_{G\text{-}R}$ values to the number of colocalized r-CAM copies. They established that the maximum $CC_{G\text{-}R}$ value at saturation occupancy was limited by the assembly stoichiometry (Fig. S1, *A–C*).

The measurement of frequencies (f_{ON},f_{OFF}) and residence time (τ) distributions from the analysis of single spots (Fig. 4) developed the ATP dependence reported by the $CC_{G\text{-}R}$ values. The f_{ON}, f_{OFF} frequencies were obtained directly from the spot population (n = 50–100) sampled over multiple records. The k_τ values were computed from exponential fits to the $|(\tau)|$ distribution for the same populations. The fits were limited to $|\tau| < 30$ s since photobleaching dominates step intensity drops for $|\tau| > 30$ s.

The r-CAM (τ) distributions for the β monomer, α dimer, and β holoenzyme are shown in Fig. 5 *A*. The monomer N_{CAM} is limited to 0–1. The residence times for zero occupancy are not scored, so the distribution should consist entirely of τ_+ events as largely observed. There were a few τ_- events. These τ_- events could denote two or more monomers with overlapping point spread functions that could not be resolved. The dimer τ distribution (N_{CAM} (0–2)) at 300 nM r-CAM was roughly symmetrical around $\tau = 0$. This implied that N_{CAM} = 2 was the dominant occupancy at this concentration. The τ_- fraction was dominant relative to the τ_+ fraction for the β holoenzyme population (N_{CAM} (0–14)), as reflected in the ($\sum n_+ / \sum n_-$) ratio.

The frequencies and rates are tabulated in Fig. S1 *D*. The monomer r-CAM k_τ rates, like the CC value, did not depend on ATP. The monomer $A_i = 300 \times k_\tau / f_{ON} = 4.1$ μM. The k_τ for the monomer distributions was 0.7 s^{-1}, independent of ATP. There was a slight (less than twofold) increase in the ATP-dependent f_{ON} frequency of the previously characterized α dimer (18) relative to the β monomer, accompanied by a striking fivefold decrease in the k_τ at 300 nM r-CAM. The k_τ rates for the α (0.59 s^{-1}) and β (0.53 s^{-1}) dimers were comparable at 30 nM r-CAM. The f_{ON} values were also similar (α [0.03 s^{-1}], β [0.07 s^{-1}]). At 30 nM r-CAM, the fit to the α dimer τ distribution was markedly biexponential, indicating two kinetically distinct populations. The heterogeneity could reflect the two different occupancy states. Only the N_{CAM} = 2 occupancy state is competent for T287 transphosphorylation. The fraction of this state will increase at higher concentrations with a corresponding increase in residence half-life ($|\tau|_{1/2}$) as observed (Fig. 5, *B* and *C*).

We conclude, first, that ATP does not alter the r-CAM accessibility or affinity for the monomeric KD CAM binding motif. Second, ATP increases the r-CAM affinity upon dimer formation. The ATP dependence of the dimer assemblies was not sensitive to linker length and other differences between the two isoforms. Third, the heterogeneity in the α dimer population at 30 nM is reduced at 300 nM r-CAM,

with the transition of the |τ| distribution from a biexponential to a longer-lived monoexponential form.

The r-CAM association in the β dimer and holoenzyme

In ATP, the r-CAM k_τ was lower by less than twofold (0.315 s^{-1}) after correction for photobleaching, while the f_{ON} decreased slightly (0.045 s^{-1}) for the β holoenzyme relative to the β dimer. The former should have had a sevenfold increase in k_{ON} if it were proportional to the number of binding motifs available in the tetradecameric holoenzyme. This fact, together with the low values, implies that the association is motif-access rather than diffusion-limited (40). The r-CAM k_τ rate decrease upon the dimer → holoenzyme transition suggests additional positive cooperativity between the holoenzyme subunits over and above that required for transphosphorylation (Fig. 5 *C*).

Finally, we assessed the N_{CAM} occupancy of the β holoenzyme population (Fig. 5 *D*) to understand the difference between the dimer and holoenzyme (τ) distributions in ATP (Fig. 5 *A*). The β holoenzyme has subsaturation r-CAM occupancy ($\overline{N_{CAM}} = 2.6 \pm 0.3$) at 30 nM r-CAM. The N_{CAM} distribution was fit by a single exponential. We checked whether the ($N_{CAM} = 0$) subpopulation represented smaller V-CaMKII assemblies. The V-CaMKII subunit stoichiometries (S_{CaMKII}) were matched to the (N_{CAM}) estimated for single-spot records and the S_{CaMKII} distribution for the ($N_{CAM} = 0$) subpopulation compared against that for the ($N_{CAM} > 0$) subpopulation distribution to test this possibility. No significant difference in the mean values was observed (Fig. 5 *D*, *inset*), validating the intensity filters set by the spot finder algorithm. The holoenzyme A_i was about 30-fold lower than that measured for the monomer, consistent with the multiple occupancy/holoenzyme. Down transitions to lower nonzero occupancy levels due to photobleaching account for the greater abundance of τ_- relative to τ_+ intervals in the holoenzyme (τ) distribution.

CAM association with engineered inactive and active β holoenzymes

Inactive and active holoenzymes have distinct CAM occupancy states

The simultaneous measurements of holoenzyme assembly state, rates, and occupancy possible in our assay established that the 30-fold ATP-dependent increase in r-CAM affinity required dimer formation. There was a further, modest 2-fold increase for holoenzymes. The extent of the increase was >100-fold lower than that reported for the trapped CAM state (13). We studied mutants in the CaMKIIβ ATP binding site (K43R) and autophosphorylation sites (T287A, T287D, T287.T306.307A) (Fig. 1) to better understand the ATP dependence. The substitutions were defined as "inactive" based on the lack of (T287A, T287.306-307A) or impaired (K43R (41)) primary site T287 autophosphorylation. The T287D substitution was defined as "active" as it is a mimic of PhosphoT287.

We first evaluated the concentration dependence of r-CAM colocalization. Colocalization for the inactive holoenzymes was measured at 30 nM r-CAM concentration where the multiple N_{CAM} occupancy and kinetics could be well-resolved from nonspecific r-CAM attachment to the glass coverslip. Although colocalization could still be detected at a 10-fold lower concentration (3 nM). In contrast, substantial T287D r-CAM colocalization was evident down to subpicomolar concentrations. All holoenzyme populations had heterogeneous subunit stoichiometry. The T287A and T287D populations, in particular, had a marked dimer fraction. Aggregation was notable in the latter populations. In the absence of ATP both the T287D dimer fraction and aggregation were reduced (Fig. S2 *A*). While aggregates could be rejected based on size, the separation of holoenzymes from smaller assemblies based on intensity filtration of the spots was critical. Comparable subunit stoichiometry (S_{CaMKII}) was achieved across all mutant populations postfiltration.

In principle, multiple factors may contribute to the subunit heterogeneity: intrinsic lability (7), a dark fluorescent protein fraction (42), double steps due to "bunching" (43), and the limited temporal resolution of the step-finder algorithm. A 20% dark fraction has been estimated for GFP (42). Inspection of individual records reveals that double steps occur predominantly at early times where steps would be most closely spaced, while simulations show our algorithm will underestimate 14-subunit holoenzyme stoichiometry by 2 ± 1 subunits (14 ± 7%) due to noise (Appendix-2). The broader range of the observed heterogeneity, together with the notable increase in the dimer fraction upon ATP-induced disassembly of the T287D holoenzyme indicates that intrinsic lability (7) is the dominant cause of the heterogeneity. The r-CAM concentrations for measurement of the occupancy of the inactive and active holoenzymes differ by 10^2- to 10^3-fold, so the <20% bleached r-CAM fraction is not consequential for the relative comparison.

The N_{CAM} mean values and distributions were then determined for the filtered populations. In the absence of ATP N_{CAM} occupancy levels comparable with the holoenzyme subunit stoichiometry were reached for the T287D holoenzyme populations at subpicomolar r-CAM. In the presence of ATP, a lower T287D N_{CAM} occupancy was obtained at a higher r-CAM concentration. In all cases, the holoenzyme-bound N_{CAM} populations were well fit by the Poisson distribution. The form of the Poisson fit changed from a skewed to the bell-shaped, normal profile as the N_{CAM} mean increased in line with expectations (Fig. 6 *A*). The fits can be compared with the ($CC_{G\text{-}R}$) values for the mutant holoenzymes at the examined r-CAM concentrations (Fig. S2 *B*). The holoenzyme Poisson distributions are

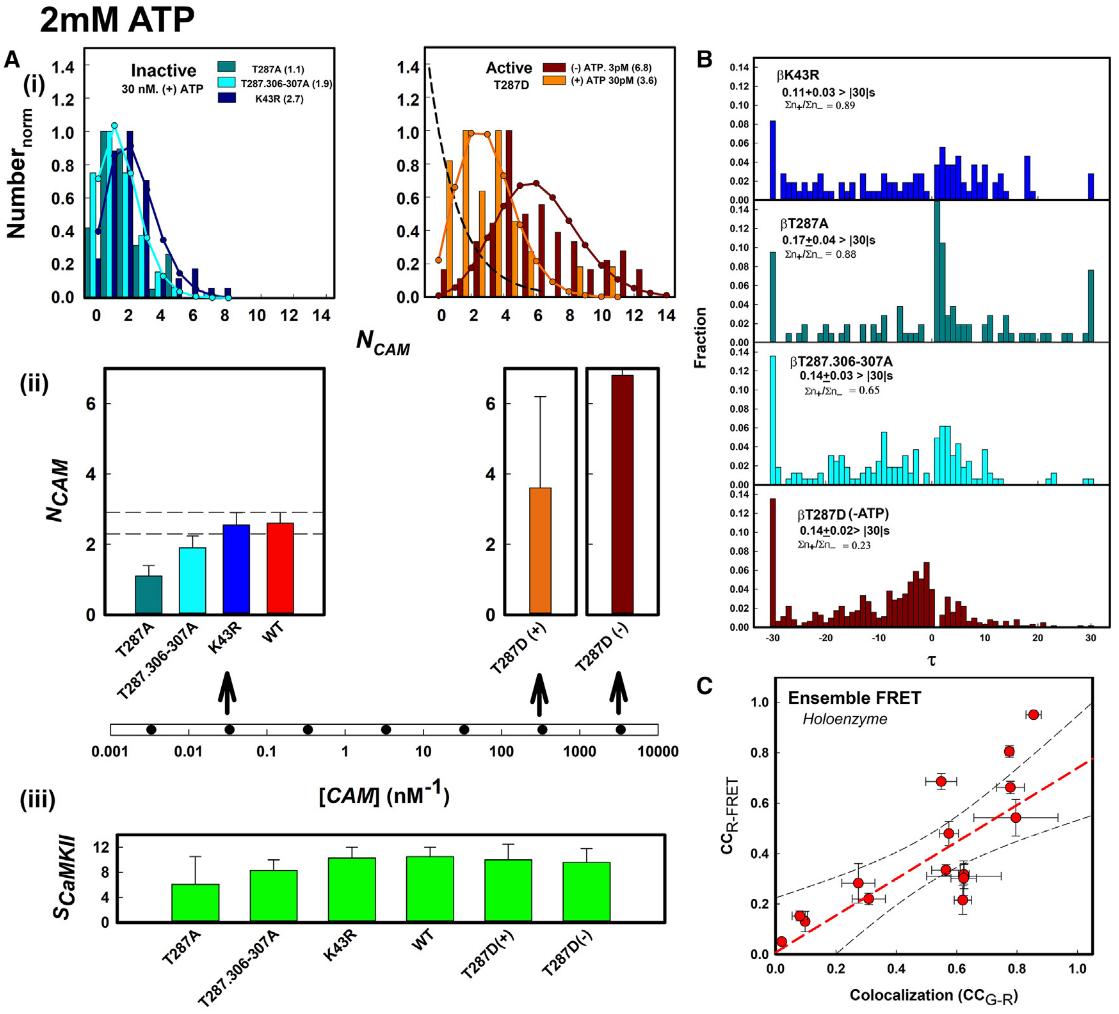

FIGURE 6 r-CAM association with mutant β holoenzymes. (*A*) r-CAM concentration dependence. (*i*) r-CaM occupancy (N_{CAM}) distributions for the T287A, T287.306.307A, and K43R silencing (inactive) holoenzymes (*left*). The T287D constitutively active holoenzyme with (+) and without (−) 2 mM ATP (*right*). Poisson fits (*solid lines*) are color coded to match the sample. (*ii*) $\overline{N_{CAM}}$ values for the inactive holoenzymes compared with the WT β holoenzyme (*left*). ATP dependence of the T287D $\overline{N_{CAM}}$ values (*right*). (*iii*) Holoenzyme subunit stoichiometries (S_{CaMKII}) determined by step photobleaching. (*B*) Histograms. CaMKII mutant residence time (τ) distributions over the −30 to +30 s range. Based on the ransom selection of >50–100< spots (2 or more experiments, >3 records/experiment). Record duration = 40 s. The step population size (Σn) = 105 (β-T287A), 286 (β-K43R), 409 (β-T287.306-307A), 183 (β-T287D [+ATP]), 933 (β-T287D [−ATP]). (*C*) FRET. All holoenzyme samples. Each data point was based on 10–20 video records/experiment (30–50 holoenzyme spots/record). There is a linear (mean [*red line*], 95% confidence limits [*gray lines*]) increase in the FRET signal ($CC_{R\text{-}FRET}$) with increased colocalization ($CC_{G\text{-}R}$). The regression coefficient = 0.93. To see this figure in color, go online.

consistent with independent binding events characterized by a single rate-limiting step for CAM association.

We next compared the wild-type (WT) holoenzyme (τ) and N_{CAM} distributions in the light of the mutant holoenzyme data. At 30 nM r-CAM (2 mM ATP), the τ distributions for the inactive holoenzymes have (τ_+/τ_-) ratios (T287A(1.76), K43R(0.89), T287.306-307A(0.65) (Fig. 6 *B*) that are elevated relative to the WT holoenzyme. In contrast, the T287D (τ_+/τ_-) ratio (0.23) at 3 pM r-CAM is lower than that obtained at 30 nM for the WT holoenzyme. The T287D r-CAM colocalization $CC_{G\text{-}R}$ values also did not vary notably with r-CAM concentration indicating close to saturation occupancy in the 0.1–100 pM r-CAM concentration regime in line with their high N_{CAM} levels.

The WT mean N_{CAM} value was a close match with that for K43R, but the single Poisson fit was not as good by comparison. A two-Poisson distribution gave an improved fit similar to those obtained for the mutant populations. The two mean values, set as floating parameters, matched the values for the low- and high-affinity N_{CAM} states recorded for the inactive and active mutant populations, respectively (Fig. S2, *C* and *D*).

The full-frame $CC_{R\text{-}FRET}$ values between the rhodamine and FRET channels, plotted against the $CC_{G\text{-}R}$ values strengthened the case for complex formation (Fig. 6 *C*).

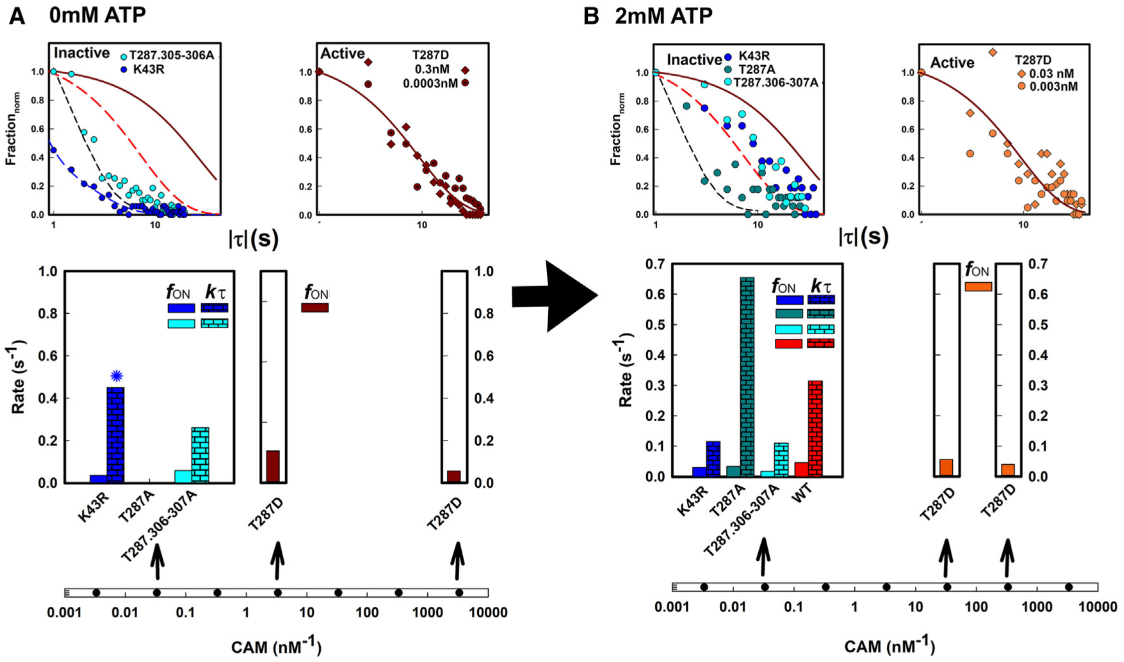

FIGURE 7 r-CAM association/dissociations for mutant β holoenzymes (−/+ATP). (*A*) 0 mM ATP. (*i*) Residence time $|(\tau)|$ distributions. K43R exponential fit (*blue dashed line*). (*ii*) f_{ON} and k_τ rates. The asterisk indicates biexponential fit. The first exponential ($k_{\tau 1} = 3.18\ s^{-1}$) is not shown. (*B*) 2 mM ATP. (*i*) Residence time $|(\tau)|$ distributions. (*ii*) f_{ON} and k_τ rates. Dashed lines show fits, and the solid red lines the expected photobleaching rate as in Fig. 5. f_{ON} (*clear*), k_τ (*brick*). To see this figure in color, go online.

Weak temporal correlations detected in the smaller monomer and dimer assembles were lost in the holoenzyme records. The reasons are explained with data and simulations in Appendix-3.

The r-CAM association/dissociation kinetics from inactive holoenzymes

The $|\tau| < 30$ s distributions for the K43R, T287A, and T287.306-307A were analyzed further to obtain the photobleaching corrected k_τ rates (Fig. 7). All kinetic parameters for the mutant holoenzymes are tabulated in Fig. S2 *E*, with plots that show the effect of the photobleaching correction on the inactive holoenzyme distributions (Fig. S2 *F*). We compared the distributions in 0 mM and saturating (2 mM) ATP to dissect the ATP dependence observed for the WT holoenzyme. In the absence of ATP, the K43R distribution was biexponential, with a fast component $k_\tau = 3.18\ s^{-1}$ that decayed more rapidly than the monomer distribution. This result demonstrates that modification of the ATP binding pocket directly affects the CAM binding site. Interestingly, at 2 mM ATP the K43R $|\tau|$ distribution overlapped with that of the WT holoenzyme. This result implies that substantial occupancy is achieved at saturating ATP for both the K43R-impaired and WT ATP binding pockets. ATP once bound to either pocket affects CAM affinity similarly. The increase did not occur if ADP was added instead of ATP, implying that the γ-phosphate or hydrolysis is required for the observed ATP dependence. Our result is in line with T286 autophosphorylation in the K42R mutant in saturating ATP (41).

We next evaluated the CaM affinity for T287.306-307A holoenzyme populations in 0 and 2 mM ATP. In 0 mM ATP the k_τ was lower than measured for the monomer (Fig. 5 *B*), but not significantly so. This result shows that holoenzyme formation per se does not affect the CAM affinity change between the native monomer and holoenzyme in the absence of ATP. In 2 mM ATP, the k_τ for the T287.306-307A holoenzyme is indistinguishable from the values computed for the K43R holoenzyme. The T287.306-307A result establishes that the K43R ATP dependence is independent of either T287 and/or T306-307 autophosphorylation.

We asked whether this ATP-dependent increase in CAM affinity could be reduced when the T306-307 residues were left unchanged to allow inhibitory autophosphorylation. T287A holoenzyme populations had markedly increased r-CAM dissociation in comparison with either WT or other inactive holoenzymes at 30 nM r-CAM (Fig. 7). The T287A τ distribution was similar to the 0 mM ATP T287.306-307A τ distribution, indicating that the ATP-dependent increase is reversed. In addition, the results, taken together (Fig. S2 *E*), show that changes in CAM affinity are largely mediated through changes in the dissociation rates, as reported previously (14).

The WT holoenzyme data may now be evaluated in light of what we have found for the inactive holoenzymes. The WT ATP dependence is mechanistically distinct from that of the T287.307–307A holoenzymes in that the T287 and

T307-307 residues would be autophosphorylated in ATP. Phosphorylation of the T306-307 residues inhibits T287 transphosphorylation, as the probability that CAM will bind to adjacent subunits is decreased. Therefore, transphosphorylation will be reduced since it requires the capture of the undocked R segment by an adjacent subunit whose own segment has been undocked. Thus, a step increase in CAM as administered in our assays could lead to a steady-state subsaturation T287 phosphorylation balanced by T306-307 phosphorylation. The result would be a subsaturation N_{CAM} value as observed.

The active T287D substitution replicates the CAM-trapped state

The use of the phosphomimetic βT287D residue substitution as an equivalent for saturating T287 phosphorylation seemed justified by its high N_{CAM} occupancy at picomolar r-CAM concentrations. In addition, the asymmetric τ distribution suggested a strong contribution of photobleaching to the apparent dissociation (Fig. 6). We therefore compared the fits to the $|\tau|$ distributions with the photobleaching expected for the recorded N_{CAM} occupancy levels to more precisely evaluate the photobleaching contribution (Fig. 7). The $|\tau|$ distributions superimpose with the predicted distribution due to photobleaching alone. While N_{CAM} occupancy is reduced in 2 mM ATP (Fig. 6), presumably due to inhibitory T306-307 phosphorylation, the reduction in the mean residence time, if any, is not detected. We conclude that dissociation from T287D holoenzymes cannot be measured in our assay due to photobleaching. The residence times measured for trapped Ca^{2+}.CaM in α holoenzymes due to T286 phosphorylation are many tens of seconds (13). Therefore, the T287D holoenzyme has all the hallmarks of the phospho-T286 α holoenzyme reported in the literature.

CONCLUSIONS

The ATP dependence of the priming reaction, the CAM association, for CaMKII kinase activation has been investigated thus far by bulk phase assays (10,13,14). Here, this reaction is investigated for the first time with a single-molecule binding assay. The time-resolved analyses of bound ligand occupancy and dwell-time distributions matched to single CaMKII assembly states, essential for mechanistic analysis of multisubunit assemblies, were inaccessible to previously used assays. In addition, subunit cooperativity may be substantially underestimated in bulk assays, the bacterial flagellar motor being a classic example (44).

We have shown that the holoenzyme CAM occupancies follow the Poisson distribution. While there are several mechanistic CaMKII models (6,45–51), a complete accounting of the topology of the CaMKII holoenzyme is prohibitive due to combinatorial complexity. A rules-based model (52) is the most complete thus far. The Poisson distribution adds another rule to guide the development of mechanistic models. Our results validate the coupling between the ATP binding pocket and the CAM binding site predicted by the dynamic network model (8). Other experimental studies have documented the reciprocal effects of bound nucleotide and substrate affinities (33–36). The transition from a monomer to a multimeric architecture affects the substrate NR2B affinity change (53), but the difference between dimer and holoenzymes was not explored in contrast to the study of the CAM affinity reported here.

The KDs are organized as catalytic domain pairs in the autoinhibited CaMKIIα holoenzyme (54). Catalytic pair formation accompanies dimerization (18). Binary FRET measurements have shown that catalytic pairs are stable for hours but separate within 5 s upon CAM association, before binding substrate (24). Binary FRET of αK2R reveals unpairing and transient binding of substrate, with subsequent dissociation accompanied by transition to another paired state. The K43R effect reported here documents modification of the ATP binding site affects CAM association independent of nucleotide occupancy.

Our study has identified two ATP-dependent states (Fig. 8). Dimerization creates an ATP-dependent state-1 for r-CAM association. The r-CAM affinity of this state is increased >30-fold relative to the monomer affinity upon assembly of the β holoenzyme. This study extends the equivalence of the dimer and holoenzyme assemblies, first measured for α isoform substrate phosphorylation (18), to CAM association. There is a modest increase in CAM affinity for the β holoenzyme relative to the β dimer for the β isoform, possibly due to the coupled KD dynamics mediated by the holoenzyme hub (55). State-1 is characterized by (20–30%) r-CAM occupancy of the WT holoenzymes at 30 nM. The phosphorylation null mutant (T287.306-307A) demonstrates that this affinity increase is independent of autophosphorylation. T306-307 phosphorylation inhibits state-1 r-CAM association as inferred from the increased T287A r-CAM k_τ rate, but a compensatory increase in T287A k_{ON}, for reasons presently unknown, reduces the r-CAM affinity difference between T287.T306-307A and T287A.

In the absence of ATP, the βT287.306-307A holoenzyme k_τ is comparable with the monomer, while the βK43R holoenzyme k_τ is higher. Allosteric coupling between the ATP and CAM binding sites seems to be responsible for state-1, as noted above. The βK43R $|\tau|$ distribution identified a subpopulation responsible for the elevated rate. This population could reflect the steric disruption to the ATP binding site, and the coupled CAM affinity, caused by the K43R substitution. In saturating ATP, our measurements show that the K43R and T287A substitutions weaken but do not block r-CAM association. Results obtained when these substitutions are used as tools in live-cell and behavioral assays, without accompanying readout of CaMKII conformational state, should be interpreted with caution. Binary FRET demonstrated that αK42R transiently bound substrate and αT286A does not block the binding of

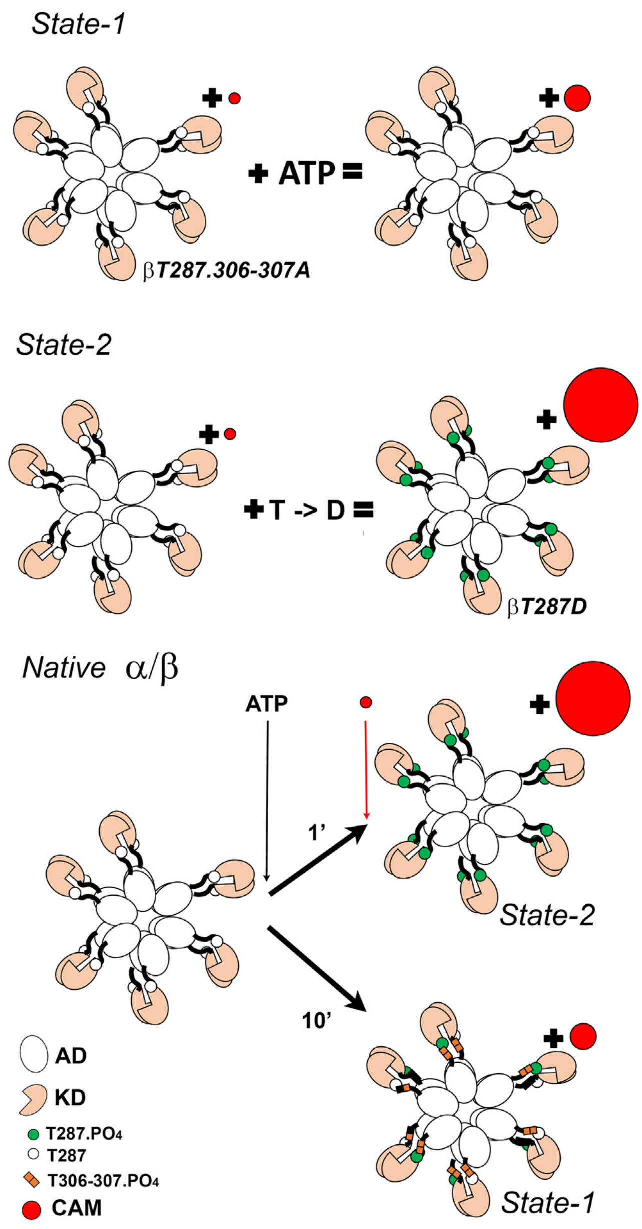

FIGURE 8 The two ATP-dependent states of the CaMKII holoenzyme. State-1 is a weak affinity state (10xmonomer-A_i). It requires dimerization and is enhanced by holoenzyme formation. This state is obtained by silent mutations of both the activating and inhibitory phosphorylation sites (T287.306-307A). Alternatively, it is obtained in the WT holoenzyme after sufficient time has elapsed for phosphorylation of the inhibitory sites. Inhibitory autophosphorylation will decrease the probability of subunit capture, hence activating transphosphorylation, to obtain a steady state of partial T287 phosphorylation. State-2 is a strong affinity state (10^4xmonomer-A_i). This state is obtained by T287D residue substitution that mimics the ATP-dependent T287 autophosphorylation. In WT holoenzymes, maximal T287 autophosphorylation (state-2) will be achieved if there is minimal inhibitory phosphorylation due to a short-time delay between ATP addition and observation. A longer time delay will allow the slower inhibitory site phosphorylation to proceed until there is a steady-state balance between activating and inhibitory phosphorylation. To see this figure in color, go online.

substrate (24). Our results extend the binary FRET data. ATP occupancy in state-1 could be important for CaMKII structural function (31).

The T287D residue substitution results in the high-affinity state-2. The state-2 picomolar affinity, $>10^4$-fold relative to the monomer affinity, matches the affinity reported for the α holoenzyme phospho-T286 state that traps CAM (13). Similar bulk assay measurements are not available for the β holoenzyme, but both isoforms have indistinguishable CAM binding isotherms ($H = 2$) (16). State-2 has near-saturation (70%) mean r-CAM occupancy comparable with the holoenzyme subunit stoichiometry at 3 pM in the absence of ATP. ATP lowers, rather than increases $\overline{N_{CAM}}$, as T306-307 can be phosphorylated in this construct. The decrease is modest, demonstrating that T306-307 autophosphorylation does not block state-2. In addition to inhibition of CAM association, there is fragmentation of the hub by T306-307 phosphorylation, so separation of the holoenzymes from smaller assemblies was important to distinguish between these effects. The T287D residue substitution has been widely used to analyze CaMKIIβ functions in live cells and neurons, but this is the first time, as far as we are aware, that this mutation has been shown to “trap” CAM, establishing it as an effective mimic of phosphorylated T287.

Why does the addition of ATP not result in saturation CAM occupancy of the WT β holoenzyme in our assay? We believe the principal factor is the dynamic balance between activating (T287) and inhibitory (T306-307) autophosphorylation important for CaMKII enzymatic function. The former reaction proceeds via intrasubunit transphosphorylation for which CAM binding to the two adjacent subunits is a prerequisite (4). As noted, the r-CAM concentration dependence of the α dimer k_τ, is consistent with this mechanism. Rapid mixing experiments have studied the interplay between activating and inhibitory phosphorylation. They reported the kinetics of α-T305-306 autoinactivation (56) and biphasic α-T286 autophosphorylation, with the slower phase thought to be due to α-T305-306 phosphorylation in the presence of CAM (3). Thus, the steady-state balance will depend on the time delay as illustrated (Fig. 8). In addition, the balance could be affected by interdomain linker length and/or in situ phosphatase access. The balance is biased toward inhibition in the human β isoform due to its long (203 residues) interdomain linker (48). Our comparison between the rat α and β dimers did not show a difference, but this could be due to a 3× shorter linker (α [31 residues], β [93 residues]) studied over a smaller (10-fold) concentration range. Interholoenzyme phosphorylation is ruled out for results obtained from our single-molecule assays, but other explanations are possible. For example, ATP could phosphorylate other sites (57–59) that were not investigated in the present study. Structural insight into holoenzyme dynamics and catalytic pair coupling will be needed to discriminate between possible mechanisms.

It has been speculated that CAM trapping by CaMKII influences frequency tuning during LTP (21). CaMKII is a cytoskeletal actin-binding protein in addition to a kinase with a pivotal role in spine remodeling due to its integrated activity (60). This study highlights the importance of spine buffer capacity, phosphatase activity, and small size on timing. Modeling suggests that Ca^{2+} influx upon synaptic stimulation would increase CAM levels within a second from nanomolar to 0.4 μM during a 100 Hz, 1-s tetanic stimulation used to induce LTP (61) The increase would achieve near-saturation CAM occupancy and autonomous T286 phosphorylation within a minute before significant T305-306 phosphorylation and mediated CAM dissociation consistent with observation (56). While phosphatases might also compete with the CAM for a common binding site (52), a minute is sufficient for CaMKII sequestration to the postsynaptic density in dendritic spines (62). In vivo FRET measurements in dendritic spines have shown that the αT287A mutation accelerated CAM dissociation, the αT305-306D mutation inhibited CAM association, and the αT286D.T306-307A mutation persistently increased CAM association (19) in qualitative agreement with our data. Continued in vitro single-molecule assays to decipher mechanism and in vivo imaging of single synapses to define relevance will be important for the elucidation of frequency decoding by CaMKII.

DATA AND CODE AVAILABILITY

The spot and step-finder ImageJ code for online acquisition and offline analysis of the videorecords has been deposited to GitHub (https://github.com/GoToJustin/Khan_et_al_2023 (63)).

SUPPORTING MATERIAL

Supporting material can be found online at https://doi.org/10.1016/j.bpj.2024.02.021.

AUTHOR CONTRIBUTIONS

S.K. conceived and executed the experiments, purified proteins, processed and interpreted the video data, assisted with code development, and wrote the manuscript. J.E.M. conceived the experiments, developed the image analysis code, assisted with analysis of the data, and wrote the manuscript. H.P. made all plasmid constructs, advised on protein expressions, and wrote the protocols. H.S. assisted with the interpretation of the data and compilation of the bibliography, and edited the manuscript. S.S.V. assisted with analysis of the single-molecule fluorescence and edited the manuscript.

ACKNOWLEDGMENTS

This study was funded by the Francis Crick Institute which receives core funding (CC119) from CRUK, MRC, Wellcome Trust (to J.E.M.) and the NIH Intramural Research Program (to S.S.V.). S.K. received travel support from the SBA School of Science & Engineering, LUMS, Pakistan.

DECLARATION OF INTERESTS

The authors declare no competing interests.

SUPPORTING CITATIONS

References (64 and 65) appear in the Supporting Material

REFERENCES

1. Zhang, M., T. Tanaka, and M. Ikura. 1995. Calcium-induced conformational transition revealed by the solution structure of apo calmodulin. *Nat. Struct. Biol.* 2:758–767.
2. Halling, D. B., B. J. Liebeskind, …, R. W. Aldrich. 2016. Conserved properties of individual Ca2+-binding sites in calmodulin. *Proc. Natl. Acad. Sci. USA.* 113:E1216–E1225.
3. Jama, A. M., J. Gabriel, …, K. Török. 2011. Lobe-specific functions of Ca2+.calmodulin in alphaCa2+.calmodulin-dependent protein kinase II activation. *J. Biol. Chem.* 286:12308–12316.
4. Bayer, K. U., and H. Schulman. 2019. CaM Kinase: Still Inspiring at 40. *Neuron.* 103:380–394.
5. De Koninck, P., and H. Schulman. 1998. Sensitivity of CaM kinase II to the frequency of Ca2+ oscillations. *Science (New York, N.Y.* 279:227–230.
6. Chao, L. H., M. M. Stratton, …, J. Kuriyan. 2011. A mechanism for tunable autoinhibition in the structure of a human Ca2+/calmodulin-dependent kinase II holoenzyme. *Cell.* 146:732–745.
7. Chien, C.-T., Puhl, H., … W. Chiu. 2023. Hub stability in the calcium calmodulin dependent protein kinase II. Under review.
8. Pandini, A., H. Schulman, and S. Khan. 2019. Conformational coupling by trans-phosphorylation in calcium calmodulin dependent kinase II. *PLoS Comput. Biol.* 15, e1006796.
9. Huse, M., and J. Kuriyan. 2002. The conformational plasticity of protein kinases. *Cell.* 109:275–282.
10. Rosenberg, O. S., S. Deindl, …, J. Kuriyan. 2005. Structure of the autoinhibited kinase domain of CaMKII and SAXS analysis of the holoenzyme. *Cell.* 123:849–860.
11. Brocke, L., M. Srinivasan, and H. Schulman. 1995. Developmental and regional expression of multifunctional Ca2+/calmodulin-dependent protein kinase isoforms in rat brain. *J. Neurosci.* 15:6797–6808.
12. Tombes, R. M., M. O. Faison, and J. M. Turbeville. 2003. Organization and evolution of multifunctional Ca(2+)/CaM-dependent protein kinase genes. *Gene.* 322:17–31.
13. Meyer, T., P. I. Hanson, …, H. Schulman. 1992. Calmodulin trapping by calcium-calmodulin-dependent protein kinase. *Science (New York, N.Y.* 256:1199–1202.
14. Singla, S. I., A. Hudmon, …, H. Schulman. 2001. Molecular characterization of calmodulin trapping by calcium/calmodulin-dependent protein kinase II. *J. Biol. Chem.* 276:29353–29360.
15. Waxham, M. N., A. L. Tsai, and J. A. Putkey. 1998. A mechanism for calmodulin (CaM) trapping by CaM-kinase II defined by a family of CaM-binding peptides. *J. Biol. Chem.* 273:17579–17584.
16. Gaertner, T. R., S. J. Kolodziej, …, M. N. Waxham. 2004. Comparative analyses of the three-dimensional structures and enzymatic properties of alpha, beta, gamma and delta isoforms of Ca2+-calmodulin-dependent protein kinase II. *J. Biol. Chem.* 279:12484–12494.
17. Nguyen, T. A., P. Sarkar, …, S. S. Vogel. 2015. Covert Changes in CaMKII Holoenzyme Structure Identified for Activation and Subsequent Interactions. *Biophys. J.* 108:2158–2170.
18. Sarkar, P., K. A. Davis, …, S. S. Vogel. 2017. Deciphering CaMKII Multimerization Using Fluorescence Correlation Spectroscopy and Homo-FRET Analysis. *Biophys. J.* 112:1270–1281.

19. Chang, J. Y., Y. Nakahata, …, R. Yasuda. 2019. Mechanisms of Ca(2+)/calmodulin-dependent kinase II activation in single dendritic spines. *Nat. Commun.* 10:2784.

20. Chao, L. H., P. Pellicena, …, J. Kuriyan. 2010. Intersubunit capture of regulatory segments is a component of cooperative CaMKII activation. *Nat. Struct. Mol. Biol.* 17:264–272.

21. Hanson, P. I., T. Meyer, …, H. Schulman. 1994. Dual role of calmodulin in autophosphorylation of multifunctional CaM kinase may underlie decoding of calcium signals. *Neuron.* 12:943–956.

22. Lučić, I., L. Héluin, …, A. J. R. Plested. 2022. CaMKII activity spreads by inter-holoenzyme phosphorylation. Preprint at bioRxiv. https://doi.org/10.1101/2022.2008.2003.502606.

23. Hoffman, L., R. A. Stein, …, H. S. McHaourab. 2011. Conformational changes underlying calcium/calmodulin-dependent protein kinase II activation. *EMBO J.* 30:1251–1262.

24. Nguyen, T. A., H. L. Puhl, 3rd, …, S. S. Vogel. 2022. Binary-FRET reveals transient excited-state structure associated with activity-dependent CaMKII - NR2B binding and adaptation. *Nat. Commun.* 13:6335.

25. Manning, G., G. D. Plowman, …, S. Sudarsanam. 2002. Evolution of protein kinase signaling from yeast to man. *Trends Biochem. Sci.* 27:514–520.

26. Hoffman, L., M. M. Farley, and M. N. Waxham. 2013. Calcium-calmodulin-dependent protein kinase II isoforms differentially impact the dynamics and structure of the actin cytoskeleton. *Biochemistry.* 52:1198–1207.

27. Khan, S., I. Conte, …, J. E. Molloy. 2016. Multiple CaMKII Binding Modes to the Actin Cytoskeleton Revealed by Single-Molecule Imaging. *Biophys. J.* 111:395–408.

28. Khan, S., K. H. Downing, and J. E. Molloy. 2019. Architectural Dynamics of CaMKII-Actin Networks. *Biophys. J.* 116:104–119.

29. Özden, C., R. Sloutsky, …, M. M. Stratton. 2022. CaMKII binds both substrates and activators at the active site. *Cell Rep.* 40, 111064.

30. Torres-Ocampo, A. P., C. Özden, …, M. M. Stratton. 2020. Characterization of CaMKIIalpha holoenzyme stability. *Protein Sci.* 29:1524–1534.

31. Tullis, J. E., M. E. Larsen, …, K. U. Bayer. 2023. LTP induction by structural rather than enzymatic functions of CaMKII. *Nature.* 621:146–153.

32. McClendon, C. L., A. P. Kornev, …, S. S. Taylor. 2014. Dynamic architecture of a protein kinase. *Proc. Natl. Acad. Sci. USA.* 111:E4623–E4631.

33. O'Leary, H., W. H. Liu, K. U. Bayer…, 2011. Nucleotides and phosphorylation bi-directionally modulate Ca2+/calmodulin-dependent protein kinase II (CaMKII) binding to the N-methyl-D-aspartate (NMDA) receptor subunit GluN2B. *J. Biol. Chem.* 286:31272–31281..

34. Cheriyan, J., P. Kumar, …, R. V. Omkumar. 2011. Calcium/calmodulin dependent protein kinase II bound to NMDA receptor 2B subunit exhibits increased ATP affinity and attenuated dephosphorylation. *PLoS One.* 6, e16495.

35. Török, K., A. Tzortzopoulos, …, R. Thorogate. 2001. Dual effect of ATP in the activation mechanism of brain Ca(2+)/calmodulin-dependent protein kinase II by Ca(2+)/calmodulin. *Biochemistry.* 40:14878–14890.

36. Tzortzopoulos, A., S. L. Best, …, K. Török. 2004. Ca2+/calmodulin-dependent activation and inactivation mechanisms of alphaCaMKII and phospho-Thr286-alphaCaMKII. *Biochemistry.* 43:6270–6280.

37. Tzortzopoulos, A., and K. Török. 2004. Mechanism of the T286A-mutant alphaCaMKII interactions with Ca2+/calmodulin and ATP. *Biochemistry.* 43:6404–6414.

38. Yang, H. C., M. M. Reedy, …, G. M. Strasburg. 1994. Calmodulin interaction with the skeletal muscle sarcoplasmic reticulum calcium channel protein. *Biochemistry.* 33:518–525.

39. Mashanov, G. I., and J. E. Molloy. 2007. Automatic detection of single fluorophores in live cells. *Biophys. J.* 92:2199–2211.

40. Bar-Even, A., E. Noor, …, R. Milo. 2011. The moderately efficient enzyme: evolutionary and physicochemical trends shaping enzyme parameters. *Biochemistry.* 50:4402–4410.

41. Tullis, J. E., N. L. Rumian, …, K. U. Bayer. 2020. The CaMKII K42M and K42R mutations are equivalent in suppressing kinase activity and targeting. *PLoS One.* 15, e0236478.

42. Ulbrich, M. H., and E. Y. Isacoff. 2007. Subunit counting in membrane-bound proteins. *Nat. Methods.* 4:319–321.

43. Kim, Y., H. L. Puhl, 3rd, …, S. S. Vogel. 2019. Venus(A206) Dimers Behave Coherently at Room Temperature. *Biophys. J.* 116:1918–1930.

44. Cluzel, P., M. Surette, and S. Leibler. 2000. An ultrasensitive bacterial motor revealed by monitoring signaling proteins in single cells. *Science (New York, N.Y.* 287:1652–1655.

45. Dupont, G., G. Houart, and P. De Koninck. 2003. Sensitivity of CaM kinase II to the frequency of Ca2+ oscillations: a simple model. *Cell Calcium.* 34:485–497.

46. Stefan, M. I., D. P. Marshall, and N. Le Novère. 2012. Structural analysis and stochastic modelling suggest a mechanism for calmodulin trapping by CaMKII. *PLoS One.* 7, e29406.

47. Michalski, P. J., and L. M. Loew. 2012. CaMKII activation and dynamics are independent of the holoenzyme structure: an infinite subunit holoenzyme approximation. *Phys. Biol.* 9, 036010.

48. Bhattacharyya, M., Y. K. Lee, …, J. Kuriyan. 2020. Flexible linkers in CaMKII control the balance between activating and inhibitory autophosphorylation. *Elife.* 9, e53670.

49. Pepke, S., T. Kinzer-Ursem, …, M. B. Kennedy. 2010. A dynamic model of interactions of Ca2+, calmodulin, and catalytic subunits of Ca2+/calmodulin-dependent protein kinase II. *PLoS Comput. Biol.* 6, e1000675.

50. Zhang, P., S. Tripathi, …, M. S. Cheung. 2017. Opposing Intermolecular Tuning of Ca(2+) Affinity for Calmodulin by Neurogranin and CaMKII Peptides. *Biophys. J.* 112:1105–1119.

51. Tripathi, S., Q. Wang, …, M. S. Cheung. 2015. Conformational frustration in calmodulin-target recognition. *J. Mol. Recogn.* 28:74–86.

52. Pharris, M. C., N. M. Patel, …, T. L. Kinzer-Ursem. 2019. A multi-state model of the CaMKII dodecamer suggests a role for calmodulin in maintenance of autophosphorylation. *PLoS Comput. Biol.* 15, e1006941.

53. Cheriyan, J., A. G. Mohanan, …, R. V. Omkumar. 2012. Effect of multimeric structure of CaMKII in the GluN2B-mediated modulation of kinetic parameters of ATP. *PLoS One.* 7, e45064.

54. Thaler, C., S. V. Koushik, …, S. S. Vogel. 2009. Structural rearrangement of CaMKIIalpha catalytic domains encodes activation. *Proc. Natl. Acad. Sci. USA.* 106 (15):6369–6374, Research Support, N.I.H., Intramural.

55. Khan, S. 2022. Conformational spread drives the evolution of the calcium-calmodulin protein kinase II. *Sci. Rep.* 12:8499.

56. Jama, A. M., J. Fenton, …, K. Török. 2009. Time-dependent autoinactivation of phospho-Thr286-alphaCa2+/calmodulin-dependent protein kinase II. *J. Biol. Chem.* 284:28146–28155.

57. Kim, K., G. Lakhanpal, …, K. Okamoto. 2015. A Temporary Gating of Actin Remodeling during Synaptic Plasticity Consists of the Interplay between the Kinase and Structural Functions of CaMKII. *Neuron.* 87:813–826.

58. Mukherji, S., and T. R. Soderling. 1995. Mutational analysis of Ca(2+)-independent autophosphorylation of calcium/calmodulin-dependent protein kinase II. *J. Biol. Chem.* 270:14062–14067.

59. Hanson, P. I., and H. Schulman. 1992. Inhibitory autophosphorylation of multifunctional Ca2+/calmodulin-dependent protein kinase analyzed by site-directed mutagenesis. *J. Biol. Chem.* 267:17216–17224.

60. Rangamani, P., M. G. Levy, …, G. Oster. 2016. Paradoxical signaling regulates structural plasticity in dendritic spines. *Proc. Natl. Acad. Sci. USA.* 113:E5298–E5307.

61. Zhabotinsky, A. M., R. N. Camp, …, J. E. Lisman. 2006. Role of the neurogranin concentrated in spines in the induction of long-term potentiation. *J. Neurosci.* 26:7337–7347.

62. Khan, S., Y. Zou, …, T. S. Reese. 2011. Sequestration of CaMKII in dendritic spines in silico. *J. Comput. Neurosci.* 31:581–594.

63. Molloy, J. E. 2023. https://github.com/GoToJustin/Khan_et_al_.

64. Min, W., and X. S. Xie. 2006. Kramers model with a power-law friction kernel: dispersed kinetics and dynamic disorder of biochemical reactions. *Phys. Rev. E - Stat. Nonlinear Soft Matter Phys.* 73:010902.

65. Lerner, E., A. Barth, …, S. Weiss. 2021. FRET-based dynamic structural biology: Challenges, perspectives and an appeal for open-science practices. *Elife.* 10:1–69.

Biophysical Journal, Volume *123*

Supplemental information

Real-time single-molecule imaging of CaMKII-calmodulin interactions

Shahid Khan, Justin E. Molloy, Henry Puhl, Howard Schulman, and Steven S. Vogel

#	Construct	Primer (Set)	Primer (Name)	Primer (5'-3' Sequence)
1	6His-SUMO-V-rCaMKIIβ*	Vector set:	SATVLBetaIFfor:	CGAGCTCAAGCTTCGAATTCTGCAGTCGACGGTTTAAATGATATCTTTGAAGC
			SATIFrev:	TCCACCAATCTGTTCTCTGTG
		Insert set:	VenforIF:	CCGCGCGGCAGCCATGTGAGCAAGGGCGAGGAGCTG
			VC1LinkIFrev:	CGAAGCTTGAGCTCGAGATC
2	6His-SUMO-V15-mCaMKIIα*	Vector set:	SMT3forIF:	GGATCCACCTCGTCCAATGCAG
			SATIFrev:	TCCACCAATCTGTTCTCTGTG
		Insert set:	ATVC1IFfor:	GAACAGATTGGTGGAGTGAGCAAGGGCGAGGAGC
			VAlpharevIF:	GGACGAGGTGGATCCTCAATGCGGCAGGACGGAG
3	6His-SUMO-V15-rCaMKIIβ**	Deletion set:	VBlinkerModFor:	GCCACCACAGTGACCTGCACC
			VBlinkerModRev:	GTCGACTGCAGAATTCGAAGC
4	6His-V15-rCaMKIIβ*	Deletion set:	VenforIF:	CCGCGCGGCAGCCATGTGAGCAAGGGCGAGGAGCTG
			SMT3revIF:	ATGGCTGCCGCGCGGCACCAG
5	6His-V15-rCaMKIIβ(monomeric/Δ315)**	Deletion set:	rCK2QC315F:	**TGA**TGCCCCGGTGGCCCCGTTACAG
			rCK2QC315R:	**TTA**CACACTGAAATTACGGGTGGC
6	6His-V15-rCaMKIIβ(dimeric/F458A)**	Mutagenesis	ratF458Afor:	**GCC**CACCGTTTCTATTTTGAAAACCTGC
			ratF458Arev:	GTCCATACCCTCCACCAGATTACC
7	6His-V15-mCaMKIIα(dimeric/F394A)**	Mutagenesis	mouseF394Afor:	**CGC**CATCGATTCTATTTTGAAAACTTG
			mouse394Arev:	GTCCAGGCCCTCCACCAGGTTC
8	6His-V15-rCaMKIIβ(silent/T287A)**	Mutagenesis	ratT287Afor:	**GCG**GTGGAATGCCTGAAGAAGTTTAATGC
			ratT287Arev:	CTCCTGGCGATGCATCATGCTTG
9	6His-V15-rCaMKIIβ(T287A/T306A/T307A)**&	Mutagenesis	ratT306/307Afor:	**GCTGCG**ATGCTGGCCACCCGTAATTTCAG
			ratT306/307Arev:	CAGAATGGCGCCCTTTAACTTGC
10	6His-V15-rCaMKIIβ(phosphomimic/T287D)**	Mutagenesis	ratT287Dfor:	**GAT**GTGGAATGCCTGAAGAAGTTTAATGC
			ratT287Drev:	CTCCTGGCGATGCATCATGCTTG
11	6His-V15-rCaMKIIβ(ATP-binding/K43R)**	Mutagenesis	βK43Rfor	**CGC**ATTATTAACACCAAGAAACTGAGC
			βK43Rrev	GGCGGCATATTCGTGGCCTGTAC

Table S1: Plasmid constructions. ***The In-Fusion Snap Assembly cloning system (TaKaRa Bio-USA, San Jose, CA) was used for insertions and some deletions. Larger insertions required amplification of overlapping vectors and insert PCR products as in constructs 1 and 2. Deletions required the generation of a single PCR product with overlapping ends as in construct 3-5, 10 and 11. **Q5® Site-Directed Mutagenesis Kit (New England Biolabs. Ipswich, MA) was used for all site-directed mutagenesis reactions and the deletion reaction in construct 3. &Mutagenesis was performed sequentially using construct 8 as the template for the triple mutant T287.306-307A. The rat and mouse CaMKII-ADs have 100% sequence identity.*

A

ASSEMBLY	[CaM]nM	[ATP]	CC_{MEAN}	CC_{SE}
β-Monomer	30	(-)	0.07	0.01
	300	(-)	0.12	0.005
	300	(+)	0.14	0.02
β-Dimer	30	(+)	0.2	0.1
	300	(+)	0.26	0.1
α-Dimer	30	(-)	0.04	0.01
	3	(+)	0.13	0.05
	30	(+)	0.34	0.1
	300	(+)	0.45	0.03
β-Holoenzyme	300	(-)	0.1	0.01
	30	(+)	0.59	0.04
	100	(+)	0.79	0.01
α-Holoenzyme	3	(+)	0.3	0.05

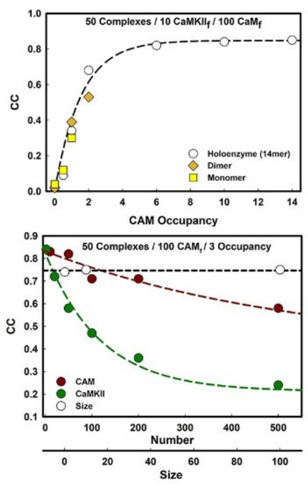

D

Construct	ATP	[r-CAM]nM	f_{ON}	f_{OFF}	$k_{\tau1}$	$k_{\tau2}$	$k_{\tau1\text{-}corr}$	A*i* nM
βmon	(-)	300	0.05	0.79	0.69			4140
βmon	(+)	300	0.042	0.79	0.69			4928.6
αdim	(+)	300	0.074	0.19	0.155			628.4
αdim	(+)	30	0.032	0.46	0.59	0.03		553.1
βdim	(+)	30	0.071	0.41	0.53			223.9
βholo	(+)	30	0.0455	0.074	0.24		0.315(0.06)	158.2

Figure S1: A. Table. Colocalization at different r-CAM concentrations (+/- ATP). *The $CC_{G\text{-}R}$ values measured colocalization. Each $CC_{G\text{-}R}$ value was based on at least 2 independent experiments, with >10 records per experiment. The typical record duration was 40 seconds (400 frames) with 30-50 spots per frame.* ***B. Figure. Simulation of $CC_{G\text{-}R}$ as a function of r-CAM occupancy.*** *The CaMKII holoenzyme tetradecamer (circle), dimer (diamond) and monomer (square). The occupancy saturates at 2 and 1 for the dimer and monomer respectively.* ***C. Figure. Simulation of $CC_{G\text{-}R}$ as a function of population size.*** *Free V-CaMKII (green circles), r-CAM (red circles) molecules and total population size (open circles). Three values for population size (1,10,100 x (50(CaMKII(14)/CAM(3) complexes), 10 free V-CaMKII, 100 free r-CAM).* ***D. Table. Apparent rate constants (s^{-1}).*** *The r-CAM concentration varied over the 1000 ->30 nM range. The monomer (βmon), dimer((αdim, βdim), and holoenzyme (βholo) assemblies. The WT β holoenzyme $k_{\tau1-corr}$ value is the photobleaching corrected $k_{\tau1}$ (Figure 4B). The photobleaching correction was not needed for the monomer or dimer assemblies. The step finder algorithm will detect spurious spots in the monomer records as the monomer intensity is not much different from background particulates and will miss ON events shorter than the algorithm's detection limit (0.5s). Both factors will underestimate f_{ON} and elevate A_i values.*

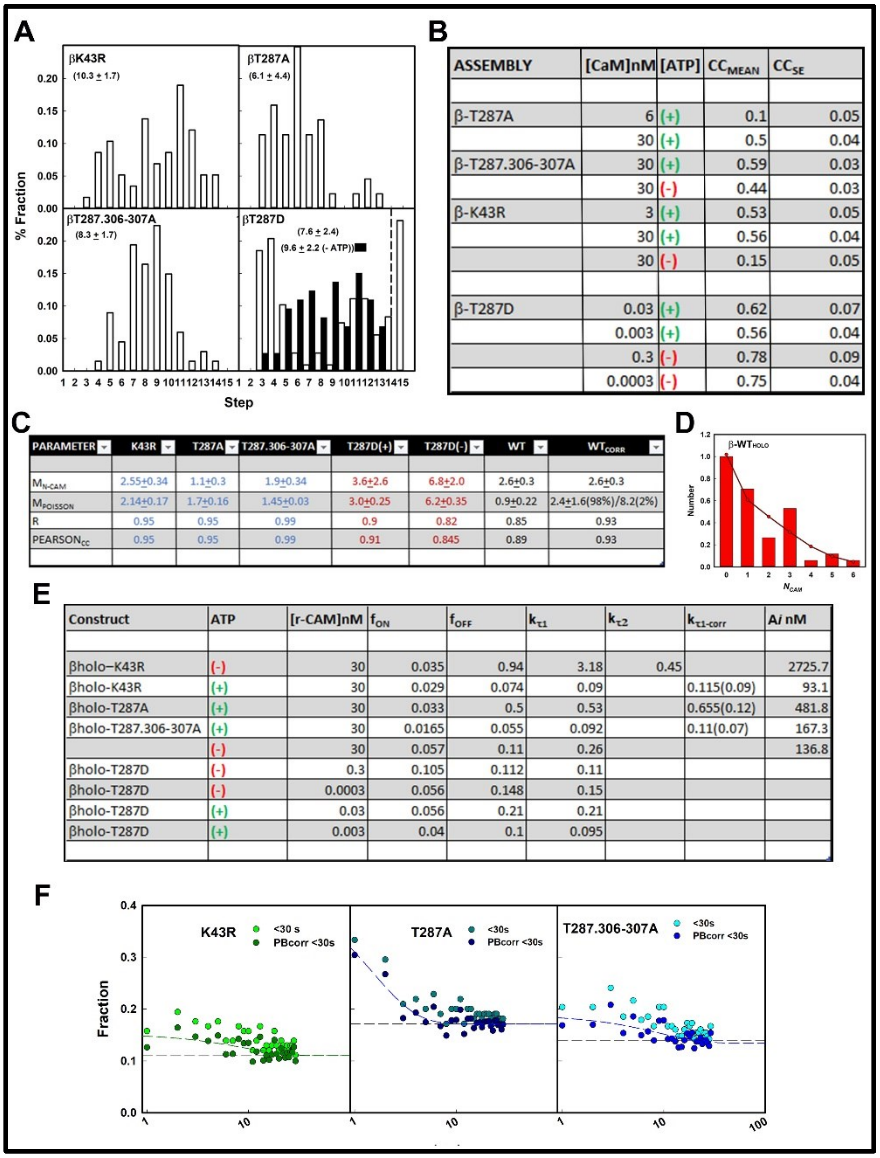

B

ASSEMBLY	[CaM]nM	[ATP]	CC_{MEAN}	CC_{SE}
β-T287A	6	(+)	0.1	0.05
	30	(+)	0.5	0.04
β-T287.306-307A	30	(+)	0.59	0.03
	30	(-)	0.44	0.03
β-K43R	3	(+)	0.53	0.05
	30	(+)	0.56	0.04
	30	(-)	0.15	0.05
β-T287D	0.03	(+)	0.62	0.07
	0.003	(+)	0.56	0.04
	0.3	(-)	0.78	0.09
	0.0003	(-)	0.75	0.04

C

PARAMETER	K43R	T287A	T287.306-307A	T287D(+)	T287D(-)	WT	WT_{CORR}
$M_{N\text{-}CAM}$	2.55±0.34	1.1±0.3	1.9±0.34	3.6±2.6	6.8±2.0	2.6±0.3	2.6±0.3
$M_{POISSON}$	2.14±0.17	1.7±0.16	1.45±0.03	3.0±0.25	6.2±0.35	0.9±0.22	2.4±1.6(98%)/8.2(2%)
R	0.95	0.95	0.99	0.9	0.82	0.85	0.93
$PEARSON_{CC}$	0.95	0.95	0.99	0.91	0.845	0.89	0.93

E

Construct	ATP	[r-CAM]nM	f_{ON}	f_{OFF}	$k_{\tau 1}$	$k_{\tau 2}$	$k_{\tau 1\text{-}corr}$	*Ai* nM
βholo–K43R	(-)	30	0.035	0.94	3.18	0.45		2725.7
βholo-K43R	(+)	30	0.029	0.074	0.09		0.115(0.09)	93.1
βholo-T287A	(+)	30	0.033	0.5	0.53		0.655(0.12)	481.8
βholo-T287.306-307A	(+)	30	0.0165	0.055	0.092		0.11(0.07)	167.3
	(-)	30	0.057	0.11	0.26			136.8
βholo-T287D	(-)	0.3	0.105	0.112	0.11			
βholo-T287D	(-)	0.0003	0.056	0.148	0.15			
βholo-T287D	(+)	0.03	0.056	0.21	0.21			
βholo-T287D	(+)	0.003	0.04	0.1	0.095			

***Figure S2: A. V-CaMKII subunit stoichiometry distributions**. The $\overline{S_{CaMKII}}$ values are compared between populations. **B. Table of colocalization coefficients ($CC_{G\text{-}R}$).** The reported $CC_{G\text{-}R}$ values were based on independent experiments with the number and duration of records analyzed similar to that for the different assemblies (Figure S1A). **C. Table.** $M_{N\text{-}CAM} = \overline{N_{CAM}}$, $M_{POISSON}$ = Mean of Poisson distribution $N_{norm}^{Poisson} = \sum_{N_{CAM}=1}^{14}(N_{CAM}\ \hat{}\ M_{POISSON})(e^{-M_{POISSON}})/N_{CAM}!)$. R = Regression coefficient, P = Pearson correlation between the fit and observed values. WT_{corr} fit equation ($N_{norm}^{WTc0rr} = a.N_{norm}^{Poisson} + (1-a)\sum_{N_{CAM}=1}^{1}\left(e^{-(N_{CAM}-M_{POISSON}^2)^2/M_{POISSON}^2)}\right)$). **D. Plot.** Fit to the WT distribution(red bars) with the WT_{corr} fit equation. The two Poisson fit has three floating parameters: the mean of the first Poisson distribution ($M_{POISSON}$), the mean of the second Poisson (Gaussian) distribution ($M_{POISSON}^2$), and "a" the amplitude fraction of the first Poisson distribution. The goodness of fit for the T287D holoenzyme N_{CAM} distribution also improved when it was fit by a two-Poisson, but the physical significance of such a fit is not presently known. **E. Table. Apparent rates (s^{-1}).** The r-CAM concentration varied over the 30nM ->0.3 pM range. The β holoenzyme (K43R, T287A, T287.305-306A) $k_{\tau 1-corr}$ values are the photobleaching corrected $k_{\tau 1}$ rates. **F. Photobleaching corrected <30s |τ| distributions.** Single exponential fits. The N_τ values are expressed relative to the value of the $N_{\tau>30}$ fraction (0 -> dashed lines).*

APPENDIX-1: Abbreviations and Operations

1. Abbreviations

ABBREVIATIONS		PARAMETERS	
r-CAM	rhodamine-tagged Ca^{2+}.CAM	N_{CAM}	r-CAM cccupancy / CaMKII
V-CaMKII	Venus-tagged CaMKII subunit	S_{CaMKII}	CaMKII subunit stoichiometry
KD	CaMKII kinase domain	f_{ON}	Frequency - ON events
AD	CaMKII association domain	f_{OFF}	Frequency - OFF events
R	CaMKII regulatory segment	$\|\tau\|$	Modulus (residence time intervals)
*CC**	Cross-correlation coefficient	τ+	Residence time intervals post ON event
P	Pearson coefficient	τ−	Residence time intervals post OFF event
A_i	Affinity index	$k_{\tau*}$	Exponent from fit to |τ | distribution (*=order)

Table A1.1: Key. *All abbreviations are defined when first used. There are three CC*'s (*$CC_{G\text{-}R}$, $CC_{G\text{-}FRET}$, $CC_{R\text{-}FRET}$*). There are two* k_{τ^*}*'s (*$k_{\tau1}, k_{\tau2}$*) for biexponential fits to the* $|\tau|$ *distribution.*

2. Spot cross-correlations (ImageJ operations)

(a) Morphological kernels for spot detection.

The 7x7 Gaussian filter

```
var mGauss = "text1=[";
 mGauss = mGauss +  "1 1 1 1 1 1 1\n";
 mGauss = mGauss +  "1 2 2 2 2 2 1\n";
 mGauss = mGauss +  "1 2 4 5 4 2 1\n";
 mGauss = mGauss +  "1 2 5 6 5 2 1\n";
 mGauss = mGauss +  "1 2 4 5 4 2 1\n";
 mGauss = mGauss +  "1 2 2 2 2 2 1\n";
 mGauss = mGauss +  "1 1 1 1 1 1 1 ]";
```

The 9x9 Laplacian of Gaussian (LoG) filter

```
var LoG = "text1=[";
  LoG += " 0  0 -1 -1 -1 -1 -1  0  0\n";
  LoG += " 0 -1 -1 -1 -1 -1 -1 -1  0\n";
  LoG += "-1 -1 -1  0  1  0 -1 -1 -1\n";
  LoG += "-1 -1  0  4  7  4  0 -1 -1\n";
  LoG += "-1 -1  1  7 10  7  1 -1 -1\n";
  LoG += "-1 -1  0  4  7  4  0 -1 -1\n";
  LoG += "-1 -1 -1  0  1  0 -1 -1 -1\n";
  LoG += " 0 -1 -1 -1 -1 -1 -1 -1  0\n";
  LoG += " 0  0 -1 -1 -1 -1 -1  0  0 ]";
```

The top 1% of detected spots were tagged with unique numbers that applied over all three channels for each spot.

b) 2D-image cross-correlation operation

```
/* This macro assumes you have two, equal sized, 32-bit images
The perimeter pixel region is set to zero
The images are cross-correlated and normalised by co-variance

The Pearson Correlation Coefficient "r" (ranges from -1 to 1)
E.g. for cross-correlation of a red and a green image:

          sum { (green[x,y] - gmean) * (red[x,y] - rmean) }
    r = -----------------------------------------------------
              std_dev {green} * std_dev {red}

Perform calculations after relative shifting the red image past the green
image

Rectangular matrix (dx,dy) = up to 16 pixels still quite fast!
        1 pixel shift in "x" up to +/-dx then
        shift down 1 pixel in "y" and repeat up to +/-dy

Store results in {dx,dy} matrix
        sum { (green[x,y] - gmean) * (red[x,y] - rmean) }
Normalise based on variance

*/
```

The cross-correlation (CC_{G-R}) between the red and green channels was measured over a 9*9-pixel^2 region. The spatial registration of the two channels was systematically shifted in single-pixel steps over a +/- 4-pixel range (in both x and y) to find the highest Pearson's coefficient.

$$CC_{G-R} = \sum_{x=-4}^{4}\sum_{y=-4}^{4}(I_{Venus} - \overline{I_{Venus}})(I_{rhodamine} - \overline{I_{rhodamine}}) /\{ \left(\sum_{x=-4}^{4}\sum_{y=-4}^{4}\left(I_{Venus} - \overline{I_{Venus}}\right)^2\right)^{0.5} * \left(\sum_{x=-4}^{4}\sum_{y=-4}^{4}\left(I_{rhodamine} - \overline{I_{rhodamine}}\right)^2\right)^{0.5} \}$$

Similar operations computed $CC_{R\text{-}FRET}$ and $CC_{G\text{-}FRET}$.

c) Excel operations

1D array correlations (Pearson (P)) and statistical tests for significance (F-test, t-test) used the available Microsoft Excel functions.

APPENDIX-2: Performance of the step-finder algorithm

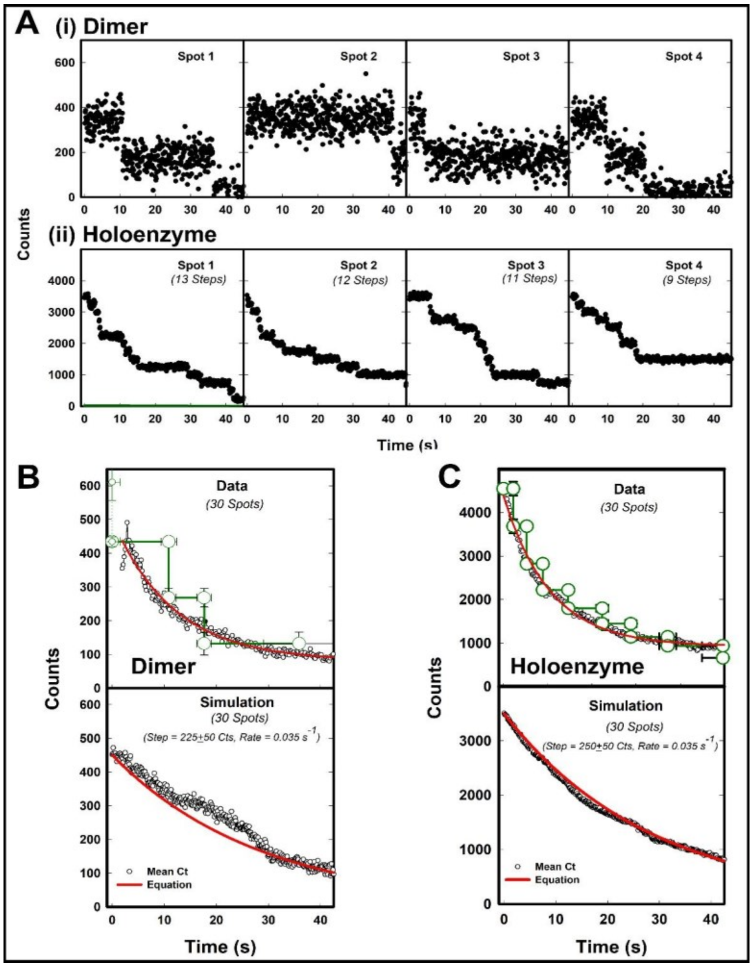

Figure A2.1. Simulation of photobleaching. A. *Examples of simulated single traces for* ***(i)*** *dimer and* ***(ii)*** *holoenzyme assemblies.* ***B.*** *Comparison of the average trace obtained from experimental versus simulated records of dimer assemblies.* ***C.*** *Comparison of the average trace obtained from experimental versus simulated records of holoenzyme assemblies. In* ***B,*** *and* ***C,*** *the observed noise and photobleaching decay exponent ($\pm$ 0.005 s^{-1}) in the experimental records was matched for the simulations. Red lines indicate both experimental fits and predicted decay. In addition to the averaged records (open circles), mean durations and amplitudes of experimental, sequential photobleaching steps are reported as stepwise drops in the mean step intensity change (green line). Green circles mark step start and end points ($\pm$ SE). The increased amplitudes of the early photobleaching events are probably due to residual errors in the subtraction of the autofluorescence background and missed short-duration steps that dominate the early record.*

The Microsoft Excel RAND() function was used to model the probability of stochastic photobleaching events, and the Gaussian-distributed noise comprising camera dark counts and photon noise as a function of spot intensity (I_0) and known EMCCD offset count value (usually ~100cts) using the transform.

$$Noise_{tot} = Offset + \left(\sqrt{I_0} + DarkNoise\right) * \sqrt{(-2 * LN(RAND()))} \; * \; COS(2\pi * RAND())$$

APPENDIX-3: The FRET signal.

The ALEX protocol was adapted from single-molecule FRET experiments (1). The amplitude of the FRET signal ordered by intensity was α-Dimer (300 > 30 nM) ~ β-monomer > β-dimer > β-holoenzyme (**Figure A3.1A**). Significant temporal correlations of the FRET signal with r-CAM colocalization were obtained for the β–monomer and dimer assemblies. The correlation for the β-dimer was worse than for the monomer. No measurable correlation was obtained for the β-holoenzyme. In all cases, the correlations were moderate. The Venus photobleaching drowned out anticipated anti-correlations between the Venus and FRET signals (**Figure A3.1B**).

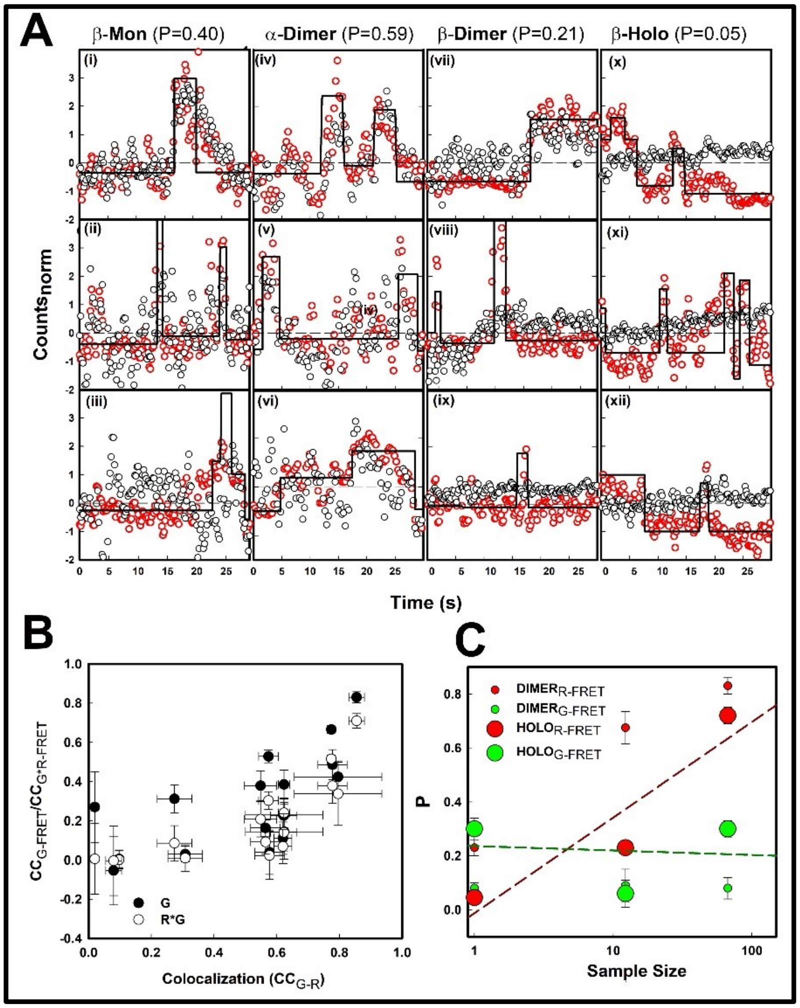

Figure A3.1: A. Temporal correlation between colocalization and FRET intensity. *Selected spot ROI records for the β monomer (**i-iii**), the α dimer (**iv-vi**), the β dimer (**vii-ix**), and β-holoenzyme (**x-xii**). Colocalization (red circles), FRET (light magenta circles). Steps reported by the step finder algorithm (black line).* ***B.*** *Correlations between the full-frame FRET versus G and G*R CCs with the colocalization ($CC_{G\text{-}R}$). The anti-correlation between the Venus and FRET signals is drowned out by Venus photobleaching The photobleaching dominates changes in the overlap integral, J.* ***C.*** *Simulations illustrate the improvement in the Pearson coefficient (P) for dimer and holoenzymes with sample size. The size ranged from single events (smallest) to a 10-spot image field (largest). The aggregate signal improved with size for the $CC_{G\text{-}R}$ versus $CC_{R\text{-}FRET}$, but not the $CC_{G\text{-}R}$ versus $CC_{G\text{-}FRET}$ correlation. This is because the $CC_{G\text{-}FRET}$ reflects Venus photobleaching, not N_{CAM} occupancy.*

The rankings can be rationalized based on the differences between linker lengths and fluorophore numbers that affect the FRET efficiency.

The FRET efficiency, $E_R = 1/\{1 + \left(\frac{r}{R_{50\%}}\right)^6\}$,

where r, the distance between the donor (Venus) and acceptor (rhodamine) within one subunit is 7±2 nm (Figure 2B). The $R_{50\%}$ is the Forster distance. The diffusion of the R peptide undocked from the KD core by bound r-CAM would supplement loop motions between the N and C-terminal KD lobes and between the tethered Venus and the N-terminal lobe to vary r for the monomer. In dimer and larger CaMKII assemblies, there will also be weaker energy transfer from Venus chromophores on adjacent subunits with the rhodamine. Their separation would vary (±4nm) depending on the fluctuations of the flexible KD-AD linkers. There is a stronger temporal correlation between the rhodamine and FRET channels for the α-dimer than the β-dimer consistent with the fact that the α-subunit has a shorter KD-AD linker (31 residues) than the β-subunit (93 residues). The fluctuations will also affect the excitation intensity profile along the z-axis of the evanescent field. The $R_{50\%}$ is 5.9 nm for the donor Venus and acceptor rhodamine-X pair. The $\left(\frac{r}{R_{50\%}}\right)$ ratio accounts for the weak FRET signals observed.

The simulation to study the dependence of the FRET signal on the Venus channel intensity fluctuations was based on the fluorescence energy transfer overlap integral, J.

The overlap integral, $J = C(R_{50\%}^6) = \int (E_D \,.\, A_A)\lambda^4 \, d\lambda$,

where E_D is donor emission, A_A is acceptor absorption. $C^{-1} = ((20.7/(128(\pi^5)N_A)) \times ((k^2)Q_D/\mu^4)$ where N_A is Avogadro's number, Q_D is quantum efficiency and m is the dielectric constant. We observe acceptor emission but assume it is proportional to its absorbance of donor photons. Residual error in the subtraction of the leakage signal (10±1.5%) would contribute to the degradation of the $CC_{G\text{-}FRET}$.

APPENDIX-4: Power fits to the holoenzyme τ distributions.

We used the exponential fit equation (Figure 4B) to correct the photobleaching rate.

$$\int_{|\tau|=30}^{30} N_\tau/N_{\tau=0} = \left((1 - A_{PB})e^{-k_{\tau-corr}t}\right) + \left((A_{PB})e^{-\overline{N_{CAM}}.k_{PB}.t}\right)$$

This equation is accurate for the monomer, and an acceptable approximation for the dimer assemblies. The photobleaching correction was not applied to these distributions, in practice, as the short τ intervals meant that photobleaching kinetics had a minimal effect in practice on the monomer and dimer r-CAM distributions. It was applied to all holoenzyme distributions (Table 2). Single exponential fits were adequate. consistent with the limited spectral data range, for comparison of the holoenzyme (τ) distributions after photobleaching correction. A further correction is needed for the fact that k also depends on N_{CAM} occupancy. The modified equation is,

$$\int_{|\tau|=0}^{30} N_\tau/N_{\tau=0} = \left((1 - (A_{PB})_{\tau=0})e^{-\overline{N_{CAM}}.k_\tau.t}\right) + ((A_{PB})_{\tau=0}.\,e^{-\overline{N_{CAM}}.k_{PB}.t})$$

This correction was also not applied in practice as the N_{CAM} occupancy was similar between the inactive holoenzymes, while no information was obtained for the active T287D holoenzyme. The mean $\overline{N_{CAM}}$ occupancy is the average over the occupancy levels for all subunits in the holoenzyme. Therefore, a complete description would take the holoenzyme subunit stoichiometry into account.

$$\int_{|\tau|=0}^{30} N_\tau/N_{\tau=0} = (A)_\tau + (A_{PB})_\tau$$

approximates a power law where

$$(A_{PB})_\tau = \int_{S_{CaMKII=1}}^{14} \int_{|\tau|=0}^{30} ((A_{N_{CAM}^{S_{CaMKII}}})e^{-N_{CAM}^{S_{CaMKII}}.k_{PB}.t})$$

$$(A)_\tau = 1 - (A_{PB})_\tau = (\int_{N_{CAM=1}}^{14} \int_{|\tau|=0}^{30} ((A_{N_{CAM}^{S_{CaMKII}}})e^{-N_{CAM}^{S_{CaMKII}}.k_\tau.t}))$$

We therefore compared exponential and power law fits to the βT287D $|\tau|$ distribution that approximated the holoenzyme photobleaching $|\tau|$ distribution at saturation N_{CAM} occupancy to better understand the complexity due to the multisubunit stoichiometry. The complete, normalized βT287D distribution is

$$(A_{PB})_{|\tau|<30} + (A_{PB})_{|\tau|>30} = 0.865 + 0.135 = 1$$

A three-exponential $((A_{PB})_{\tau-norm} = (0.1(e^{-0.2\tau}))+(0.053(e^{-0.01\tau})) + 0.1(e^{-0.07\tau}))$ fit (R=0.93) to the distribution is required to account for the $(A_{PB})_{\tau>30}$ fraction. As the number of exponents increases, multi-

exponential fits approximate a power law (2, 3). The three exponential fit is compared with a power law fit. While the fits to the < 30 s data are comparable (R=0.93), only the power law fit accounts for the > 30 s fraction (**Figure A2A**). The native and inactive holoenzyme distributions were corrected by subtraction of the inferred photobleaching events from the total events over t = 0->30s based on $(A_{kPB}{*}(e^{-k_{fPB}t}))$. The correction and single exponential fit (R=0.68) are illustrated for the native β holoenzyme (**Figure A2B**). The $k_{\tau 1-corr}$ distributions and fits for the inactive β holoenzymes (K43R (R=0.6), T287A (R=0.65), T287.306-307A (R=0.67)) are shown in **Figure A2C**. Their |τ| > 30s fractions have similar values to the βT287D fraction (0.14±0.02) since they are almost entirely due to the photobleaching of these multimeric assemblies.

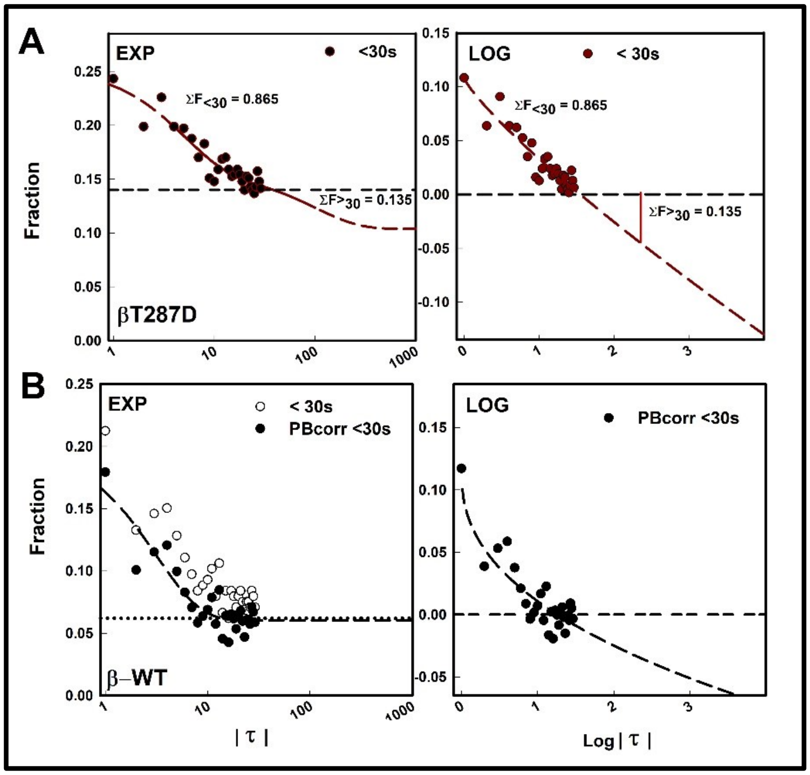

Figure A4.1: *Exponential versus power law fits for the active (βT287D) and inactive (βT287.306-307A) τ distributions.* ***A.*** *βT287D. Dashed red lines show the tri-exponential (EXP) and power (LOG) fits. Dashed black lines partition the τ <30s and >30s sub-populations. The τ <30s fraction is the area demarcated by the fit and the partition line. The τ >30s fraction is the area demarcated by the vertical red line (τ = 226s), the fit and the partition line. The tri-exponential fit will be extended well beyond τ = 226s to account for the | τ | >30s fraction.* ***B.*** *Native β. Comparison of the single exponential and power fits to the photobleaching-corrected (PBcorr) distribution.*

REFERENCES

1. Lerner, E., A. Barth, J. Hendrix, B. Ambrose, V. Birkedal, S. C. Blanchard, R. Borner, H. Sung Chung, T. Cordes, T. D. Craggs, A. A. Deniz, J. Diao, J. Fei, R. L. Gonzalez, I. V. Gopich, T. Ha, C. A. Hanke, G. Haran, N. S. Hatzakis, S. Hohng, S. C. Hong, T. Hugel, A. Ingargiola, C. Joo, A. N. Kapanidis, H. D. Kim, T. Laurence, N. K. Lee, T. H. Lee, E. A. Lemke, E. Margeat, J. Michaelis, X. Michalet, S. Myong, D. Nettels, T. O. Peulen, E. Ploetz, Y. Razvag, N. C. Robb, B. Schuler, H. Soleimaninejad, C. Tang, R. Vafabakhsh, D. C. Lamb, C. A. Seidel, and S. Weiss. 2021. FRET-based dynamic structural biology: Challenges, perspectives and an appeal for open-science practices. Elife 10.
2. Min, W., and X. S. Xie. 2006. Kramers model with a power-law friction kernel: dispersed kinetics and dynamic disorder of biochemical reactions. Phys Rev E Stat Nonlin Soft Matter Phys 73(1 Pt 1):010902.
3. Khan, S., I. Conte, T. Carter, K. U. Bayer, and J. E. Molloy. 2016. Multiple CaMKII Binding Modes to the Actin Cytoskeleton Revealed by Single-Molecule Imaging. Biophysical Journal 111(2):395-408.
